# Supplementary material for: Efficacy of the tetravalent protein COVID-19 vaccine, SCTV01E: a phase 3 double-blind, randomized, placebo-controlled trial
Source: Nat Commun. 2024 Jul 24;15:6255. doi: 10.1038/s41467-024-49832-7 (PMC11269576; doi:10.1038/s41467-024-49832-7)
Supplement: Supplementary file 1 — Supplementary Information [file 41467_2024_49832_MOESM1_ESM.pdf]

Supplementary Appendix

Table of contents

Table S1. Demographic Characteristics of Participants in the Full Analysis Set. 1
Table S2. Demographic Characteristics of Participants in Per-Protocol Set for Efficacy 4
Table S3. Vaccine Sequences of Priming and Booster Vaccine 6
Table S4. Symptoms of SARS-CoV-2 Infection in the Per-Protocol Set for Efficacy 7
Table S5. CT Values for Confirmed Primary Endpoint Cases in the Full Analysis Set 8
Table S6. Subgroup Analysis of Safety 9
Figure S1. Vaccine Efficacy Analysis on Population in the Per-Protocol Set 11
Figure S2. Genotype and Evolution of Specific SARS-CoV-2 Strains in the Full Analysis Set.....12
Figure S3. Flow Chart of the Adjudication Process of COVID-19 Cases......................................13
Figure S4. Flow Chart of Multiple Comparisons 14

Table S1. Demographic Characteristics of Participants in the Full Analysis Set.

Characteristics	Placebo (N=4595)	SCTV01E (N=4601)	Total (N=9196)	P values (Comparison between two groups)
Age (Years)				0.6646
N (Missing)	4595 (0)	4601 (0)	9196 (0)	
Mean (SD)	49.4 (14.8)	49.5 (14.8)	49.5 (14.8)	
Median	51.0	51.0	51.0	
Min, Max	18, 88	18, 95	18, 95	
Age subgroups-randomization, n (%)				0.9613
18-59 years	3471 (75.5%)	3473 (75.5%)	6944 (75.5%)	
≥60 years	1124 (24.5%)	1128 (24.5%)	2252 (24.5%)	
Sex, n (%)				0.4999
Male	2619 (57.0%)	2655 (57.7%)	5274 (57.4%)	
Female	1976 (43.0%)	1946 (42.3%)	3922 (42.6%)	
BMI (kg/m^2)				0.2755
N (Missing)	4594 (1)	4600 (1)	9194 (2)	
Mean (SD)	24.1 (3.5)	24.2 (3.5)	24.1 (3.5)	
Median	23.8	24.0	23.9	
Min, Max	15.4, 49.0	14.1, 49.9	14.1, 49.9	
Results of rapid antigen test at baseline, n (%)				0.3431
Negative	4584 (99.8%)	4595 (99.9%)	9179 (99.8%)	
Positive	6 (0.1%)	3 (0.1%)	9 (0.1%)	
Total (Missing)	4590 (5)	4598 (3)	9188 (8)	
Results of the nucleic acid test at baseline, n (%)				0.0169

Characteristics	Placebo (N=4595)	SCTV01E (N=4601)	Total (N=9196)	P values (Comparison between two groups)
Negative	3877 (84.4%)	3961 (86.1%)	7838 (85.2%)	
Positive	715 (15.6%)	634 (13.8%)	1349 (14.7%)	
Total (Missing)	4592 (3)	4595 (6)	9187 (9)	
Baseline total anti-spike IgG (BAU/mL)				0.9166
N (Missing)	4592 (3)	4599 (2)	9191 (5)	
Mean (SD)	1690.7 (1421.5)	1693.7 (1420.3)	1692.2 (1420.9)	
Median	1765.3	1784.7	1777.4	
Min, Max	6, 3200	6, 3200	6, 3200	
Stratified by baseline total anti-spike IgG, n (%)				0.8447
<338 BAU/mL	1638 (35.6%)	1650 (35.9%)	3288 (35.8%)	
≥338 BAU/mL	2954 (64.3%)	2949 (64.1%)	5903 (64.2%)	
Total (Missing)	4592 (3)	4599 (2)	9191 (5)	
Types of last received COVID-19 vaccine-randomization, n (%)				0.8121
Inactivated vaccine	3406 (74.1%)	3400 (73.9%)	6806 (74.0%)	
Non-inactivated vaccine	1189 (25.9%)	1201 (26.1%)	2390 (26.0%)	
History of COVID-19, n (%)				1.0000
Yes	0	1 (<0.1%)	1 (<0.1%)	
No	4595 (100.0%)	4600 (100.0%)	9195 (100.0%)	

Characteristics	Placebo (N=4595)	SCTV01E (N=4601)	Total (N=9196)	P values (Comparison between two groups)
Vaccination intervals (Month)				0.2575
N (Missing)	4595 (0)	4601 (0)	9196 (0)	
Mean (SD)	13.2 (3.0)	13.1 (3.0)	13.1 (3.0)	
Median	13.0	13.0	13.0	
Min, Max	6, 24	5, 24	5, 24	
Vaccination intervals, n (%)				0.5586
6-12 months	2150 (46.8%)	2181 (47.4%)	4331 (47.1%)	
>12 months	2445 (53.2%)	2419 (52.6%)	4864 (52.9%)	
Total (Missing)	4595 (0)	4600 (1)	9195 (1)	
Pre-existing comorbidities, n (%)				0.2237
Yes	967 (21.0%)	1017 (22.1%)	1984 (21.6%)	
No	3628 (79.0%)	3584 (77.9%)	7212 (78.4%)	

Descriptive statistics for continuous demographic variables include the sample size, mean, standard deviation, minimum, median, and maximum values, while categorical variables are described by sample size and percentage.

All P values in Table S1 are two-sided and descriptive in nature without adjustment for multiple comparisons. For the inter-group comparsions, T-Test and Chi-Square test were used for continuous variables and categorical variables, respectively.

Table S2. Demographic Characteristics of Participants in Per-Protocol Set for Efficacy

Characteristics	Placebo (N=1309)	SCTV01E (N=1314)	Total (N=2623)
Age (Years)			
N (Missing)	1309 (0)	1314 (0)	2623 (0)
Mean (SD)	51.2 (15.3)	51.6 (14.6)	51.4 (14.9)
Median	54.0	53.0	53.0
Min, Max	18, 88	18, 92	18, 92
Age subgroups-randomization, n (%)			
18-59 years	915 (69.9%)	928 (70.6%)	1843 (70.3%)
≥60 years	394 (30.1%)	386 (29.4%)	780 (29.7%)
Sex, n (%)			
Male	816 (62.3%)	832 (63.3%)	1648 (62.8%)
Female	493 (37.7%)	482 (36.7%)	975 (37.2%)
BMI (kg/m^2)			
N (Missing)	1308 (1)	1313 (1)	2621 (2)
Mean (SD)	24.0 (3.5)	24.0 (3.3)	24.0 (3.4)
Median	23.5	23.7	23.6
Min, Max	16.3, 49.0	16.5, 49.9	16.3, 49.9
Results of rapid antigen test at baseline, n (%)			
Negative	1309 (100.0%)	1314 (100.0%)	2623 (100.0%)
Positive	0	0	0
Results of the nucleic acid test at baseline, n (%)			
Negative	1306 (99.8%)	1314 (100.0%)	2620 (99.9%)
Positive	0	0	0
Total (Missing)	1306 (3)	1314 (0)	2620 (3)
Baseline total anti-spike IgG (BAU/mL)			
N (Missing)	1309 (0)	1314 (0)	2623 (0)
Mean (SD)	128.5 (83.5)	127.0 (82.6)	127.8 (83.0)
Median	106.3	108.7	107.7

Characteristics	Placebo (N=1309)	SCTV01E (N=1314)	Total (N=2623)
Min, Max	6, 338	6, 338	6, 338
Stratified by baseline total anti-spike IgG, n (%)			
<338 BAU/mL	1309 (100.0%)	1314 (100.0%)	2623 (100.0%)
≥338 BAU/mL	0	0	0
Types of last received COVID-19 vaccine-randomization, n (%)			
Inactivated vaccine	952 (72.7%)	971 (73.9%)	1923 (73.3%)
Non-inactivated vaccine	357 (27.3%)	343 (26.1%)	700 (26.7%)
History of COVID-19, n (%)			
Yes	0	0	0
No	1309 (100.0%)	1314 (100.0%)	2623 (100.0%)
Vaccination intervals (Month)			
N (Missing)	1309 (0)	1314 (0)	2623 (0)
Mean (SD)	13.3 (3.0)	13.3 (3.0)	13.3 (3.0)
Median	13.0	13.0	13.0
Min, Max	6, 21	7, 24	6, 24
Vaccination intervals, n (%)			
6-12 months	603 (46.1%)	583 (44.4%)	1186 (45.2%)
>12 months	706 (53.9%)	731 (55.6%)	1437 (54.8%)
Pre-existing comorbidities, n (%)			
Yes	303 (23.1%)	303 (23.1%)	606 (23.1%)
No	1006 (76.9%)	1011 (76.9%)	2017 (76.9%)

The population in the per-protocol set for efficacy included participants who had negative results of baseline nasal/nasopharyngeal/throat swab nucleic acid test, with baseline total anti-spike antibodies < 338 BAU/mL, and no SARS-CoV-2 infection within 7 days after study vaccination.

Table S3. Vaccine Sequences of Priming and Booster Vaccine

1st dose	2nd dose	3rd dose	Number	Percentage (%)		
				Total	Placebo	SCTV01E
Inactivated vaccine			3	0.03%	0	0.07%
Adenovirus-vector vector vaccine			14	0.15%	0.20%	0.11%
Protein-based vaccine	Protein-based vaccine		2	0.02%	0	0.04%
Inactivated vaccine		Inactivated vaccine	1	0.01%	0.02%	0
Inactivated vaccine	Inactivated vaccine		429	4.67%	4.50%	4.83%
Adenovirus-vector vaccine	Inactivated vaccine		1	0.01%	0	0.02%
Adenovirus-vector vaccine	Adenovirus-vector vaccine		119	1.29%	1.28%	1.30%
Protein-based vaccine	Protein-based vaccine	Protein-based vaccine	1669	18.15%	18.19%	18.10%
Protein-based vaccine	Inactivated vaccine	Protein-based vaccine	1	0.01%	0	0.02%
Protein-based vaccine	Inactivated vaccine	Inactivated vaccine	3	0.03%	0.04%	0.02%
Inactivated vaccine	Inactivated vaccine	Protein-based vaccine	524	5.70%	5.53%	5.87%
Inactivated vaccine	Inactivated vaccine	Inactivated vaccine	6321	68.74%	69.03%	68.44%
Inactivated vaccine	Inactivated vaccine	Adenovirus-vector vaccine	108	1.17%	1.15%	1.20%
Adenovirus-vector vaccine	Adenovirus-vector vaccine	Adenovirus-vector vaccine	1	0.01%	0.02%	0

Table S4. Symptoms of SARS-CoV-2 Infection in the Per-Protocol Set for Efficacy

	Placebo	SCTV01E
Duration Time (Day)		
Case	110	43
Mean (SD)	6.0 (7.8)	4.7 (8.8)
Median	3.5	1.0
Upper quartile, Lower quartile	0.0, 8.0	0.0, 6.0
Min, Max	0, 38	0, 51
P value		0.0941
Number of Symptoms		
Case	110	43
Mean (SD)	3.1 (2.6)	2.1 (1.6)
Median	2.0	1.0
Upper quartile, Lower quartile	1.0, 4.0	1.0, 3.0
Min, Max	1, 11	1, 6
P value		0.0495

The duration and number of SARS-CoV-2-infection related symptoms from participants who had been infected with SARS-CoV-2 with the onset of 7 days after the study vaccination were analyzed among the population in the per-protocol set for efficacy.

Table S5. CT Values for Confirmed Primary Endpoint Cases in the Full Analysis Set

		**Placebo (N=4595)**	**SCTV01E (N=4601)**
CDC-ORF1AB CT	Case	42	12
	Mean (SD)	29.4 (5.4)	33.1 (5.4)
	Median	28.7	34.2
	Min, Max	20.3, 38.7	23.1, 39.0
	P value		0.0426
Virus load (copies/mL)		2.3×10^4	4.8×10^2
CDC-N CT	Case	46	12
	Mean (SD)	31.0 (5.7)	33.3 (4.9)
	Median	31.5	35.3
	Min, Max	20.6, 39.7	24.0, 38.8
	P value		0.1901
Virus load (copies/mL)		3.2×10^3	2.2×10^2

CT values of the samples from participants who had confirmed symptomatic SARS-CoV-2 infection were analyzed among the population in the full analysis set who had received at least one dose of the study vaccine.

Table S6. Subgroup Analysis of Safety

	Negative results of nucleic acid/rapid antigen test at baseline		Positive results of nucleic acid/rapid antigen test at baseline	
	**Placebo (N=3879) n (%)**	**SCTV01E (N=3967) n (%)**	**Placebo (N=716) n (%)**	**SCTV01E (N=634) n (%)**
Treatment Emergent Adverse Event (TEAE)	423 (10.9)	851 (21.5)	54 (7.5)	88 (13.9)
Within 30 minutes after vaccination	47 (1.2)	104 (2.6)	9 (1.3)	11 (1.7)
Within 7 days after vaccination	311 (8.0)	745 (18.8)	39 (5.4)	72(11.4)
Within 28 days after vaccination	423 (10.9)	851 (21.5)	54 (7.5)	88 (13.9)
Any Study Vaccine-Related TEAEs	251 (6.5)	708 (17.8)	33 (4.6)	67 (10.6)
Within 30 minutes after vaccination	47 (1.2)	104 (2.6)	9 (1.3)	11 (1.7)
Within 7 days after vaccination	249 (6.4)	707 (17.8)	32 (4.5)	67 (10.6)
Within 28 days after vaccination	251 (6.5)	708 (17.8)	33 (4.6)	67 (10.6)
Any Grade 3 and above TEAEs	49 (1.3)	51 (1.3)	7 (1.0)	5 (0.8)
Any Grade 3 and above Study Vaccine-Related TEAEs	14 (0.4)	15 (0.4)	0	2 (0.3)
Any Serious Adverse Events	32 (0.8)	36 (0.9)	6 (0.8)	4 (0.6)
Within 28 days after vaccination	13 (0.3)	19 (0.5)	4 (0.6)	1 (0.2)
28 days after vaccination	19 (0.5)	17 (0.4)	2 (0.3)	3 (0.5)
Any Study Vaccine-Related Serious Adverse Events	0	0	0	0
Within 28 days after vaccination	0	0	0	0
28 days after vaccination	0	0	0	0
Solicited Local Adverse Events	136 (3.5)	622 (15.7)	23 (3.2)	57 (9.0)
Any Study Vaccine-Related Solicited Local Adverse Events	136 (3.5)	622 (15.7)	23 (3.2)	57 (9.0)
Solicited Systemic Adverse Events	182 (4.7)	271 (6.8)	10 (1.4)	20 (3.2)
Any Study Vaccine-Related Solicited Systemic Adverse Events	137 (3.5)	227 (5.7)	9 (1.3)	19 (3.0)
Un-solicited TEAEs	206 (5.3)	231 (5.8)	24 (3.4)	24 (3.8)
Any Study Vaccine-Related Un-solicited TEAEs	32 (0.8)	45 (1.1)	1 (0.1)	2 (0.3)

	Negative results of nucleic acid/rapid antigen test at baseline		Positive results of nucleic acid/rapid antigen test at baseline	
	**Placebo (N=3879) n (%)**	**SCTV01E (N=3967) n (%)**	**Placebo (N=716) n (%)**	**SCTV01E (N=634) n (%)**
Adverse Events Leading to Study Discontinuation	1 (<0.1)	0	0	0
Any Study Vaccine-Related Adverse Events Leading to Study Discontinuation	0	0	0	0
Adverse Events of Special Interest	0	0	0	0
Any Study Vaccine-Related Adverse Events of Special Interest	0	0	0	0
Death	1 (<0.1)	0	0	0

Figure S1. Vaccine Efficacy Analysis on Population in the Per-Protocol Set

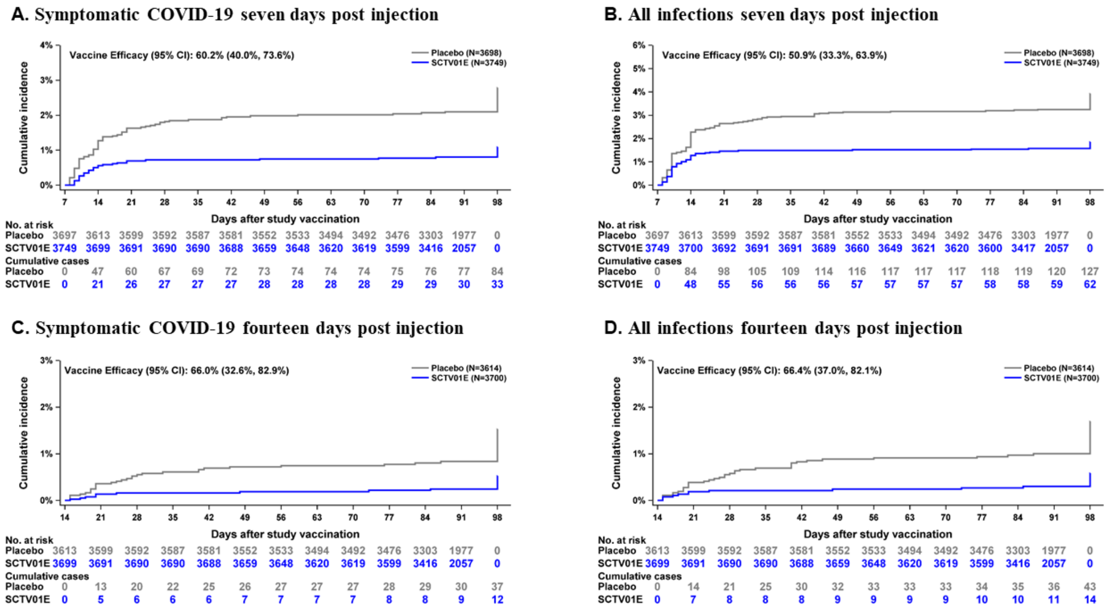

Cumulative incidence for COVID-19 cases at 7 and 14 days post-injection of SCTV01E or placebo among population in the per-protocol set were depicted in Panels A (symptomatic COVID-19 7 days post injection), B (all infections 7 days post injection), C (symptomatic COVID-19 14 days post injection), and D (all infections 14 days post injection).

Figure S2. Genotype and Evolution of Specific SARS-CoV-2 Strains in the Full Analysis Set

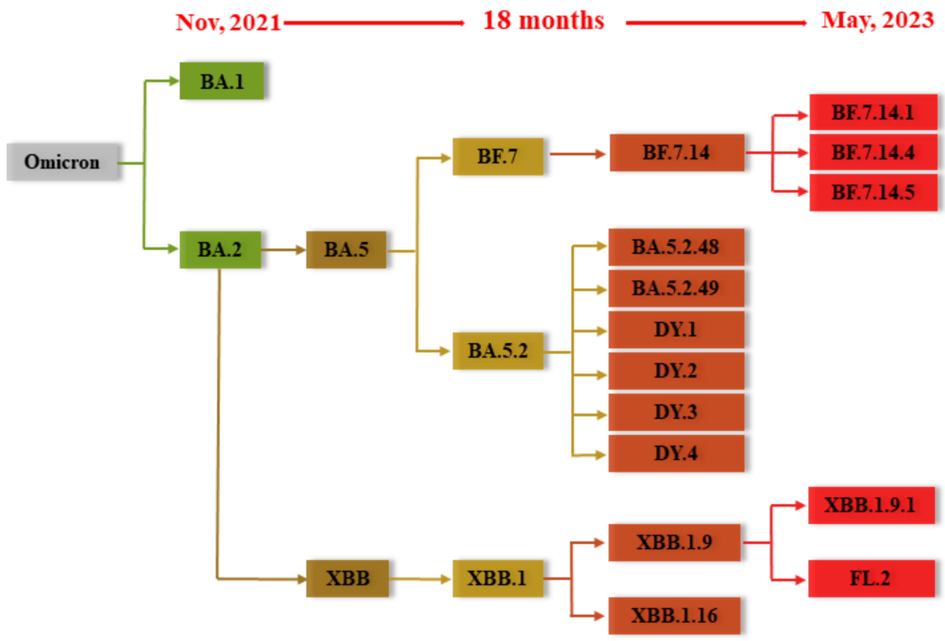

mutation / variant	D339	R346	L368	L371	T376	D405	R408	V445	S446	L452	N460	K478	F486	F490	R493	S496
BA.1																
BF.7.14		T		F	A	N	S		G	R			V		Q	G
BA.5.2.48				F	A	N	S		G	R			V		Q	G
BA.5.2.49				F	A	N	S		G	R			V		Q	G
DY.1				F	A	N	S		G	R			V		Q	G
DY.2				F	A	N	S		G	R			V		Q	G
DY.3				F	A	N	S		G	R			V		Q	G
DY.4				F	A	N	S		G	R			V		Q	G
XBB.1.16	H	T	I	F	A	N	S	P			K	R	P	S	Q	G
BF.7.14.1		T		F	A	N	S		G	R			V		Q	G
BF.7.14.4		T		F	A	N	S		G	R			V		Q	G
BF.7.14.5		T		F	A	N	S		G	R			V		Q	G
XBB.1.9.1	H	T	I	F	A	N	S	P			K		P	S	Q	G
FL.2	H	T	I	F	A	N	S	P			K		P	S	Q	G

The mutations in the RBD domain are compared to SARS-CoV-2 Omicron BA.1 variant.

Figure S3. Flow Chart of the Adjudication Process of COVID-19 Cases

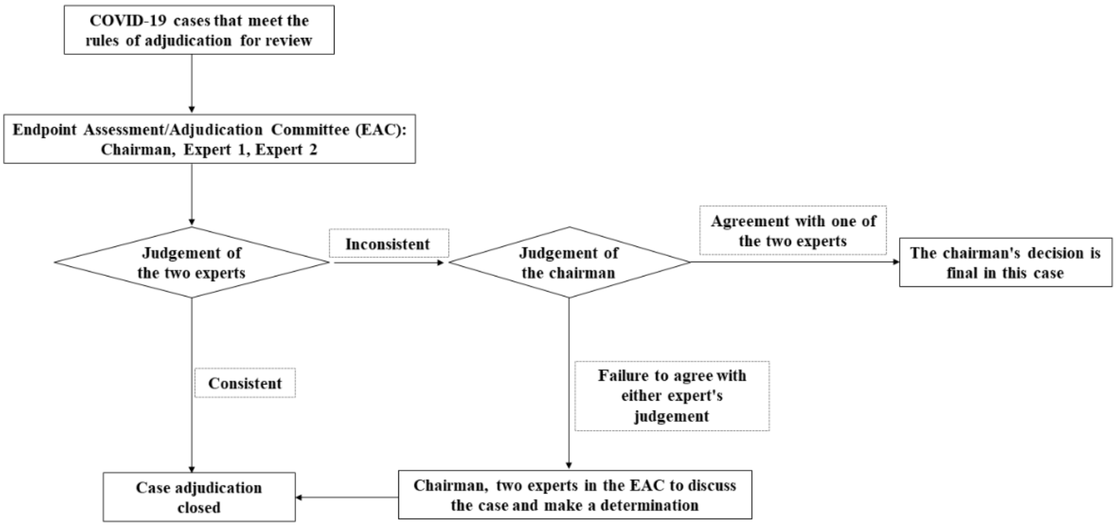

Figure S4. Flow Chart of Multiple Comparisons

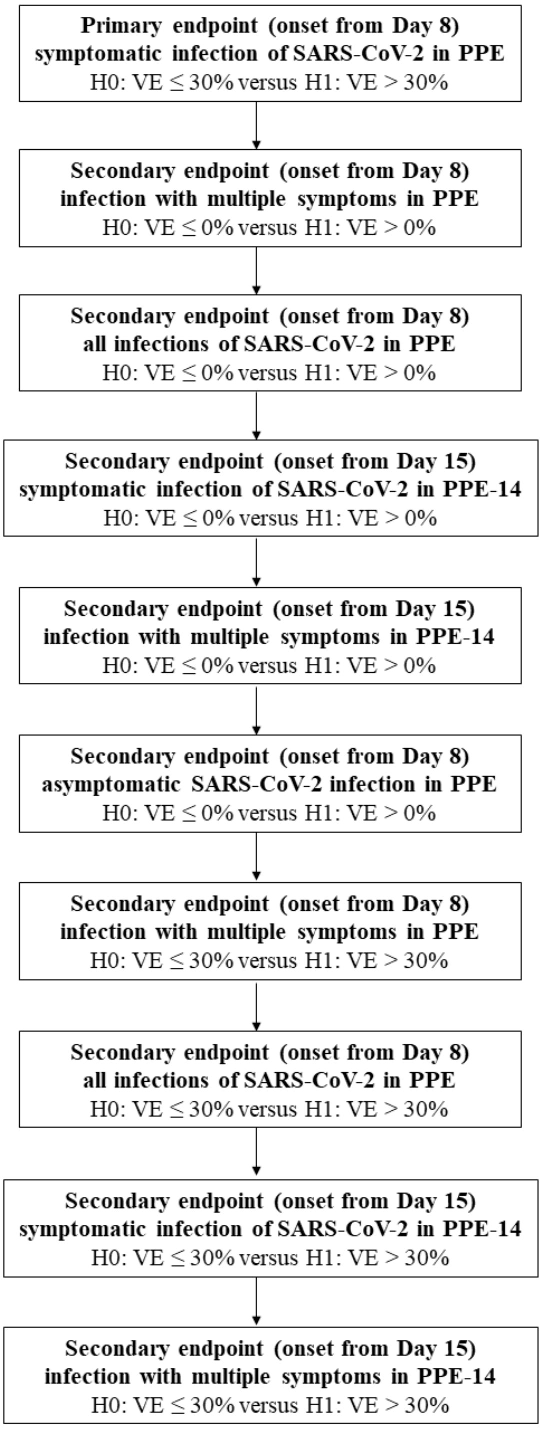

Clinical Trial Protocol

A randomized, double-blind, placebo-controlled Phase III clinical trial to evaluate the efficacy and safety of SCTV01E (A COVID-19 Alpha/Beta/Delta/Omicron Variants S-Trimer Vaccine) in healthy adults aged ≥18 years

**Protocol No.:**	SCTV01E-MRCT-2
**Protocol Version No.:**	Version 6.0
**Version date:**	May 5, 2023
**Sponsor:**	Sinocelltech Ltd.<br>31 Kechuang 7th Street, BDA, Beijing, China<br>Phone: +86-10-58628288 Fax: +86-10-5862-8299<br>Website: www.sinocelltech.com

Confidentiality Statement

All information in this protocol constitutes proprietary property of Sinocelltech Ltd. Therefore, it is only provided to investigators, co-investigators, ethics committees, regulatory authorities, and other relevant medical institutions for review. Without the written approval of Sinocelltech Ltd., it is strictly forbidden to inform any third party unrelated to this study of any information herein, except where required by applicable law. In the event of any actual or suspected breach of this obligation, Sinocelltech Ltd. must be promptly notified.

Protocol Signature Page

I agree to:

- Conduct this study in strict accordance with the protocol, quality management practices of clinical drug trials and relevant laws and regulations.
- Keep all materials and information provided by Sinocelltech Ltd. in accordance with confidentiality requirements and indicate that they are confidential when submitted to Institution Review Committee or Independent Ethics Committee.

I have read the protocol in full and agree with all requirements.

______________________	______________________	______________________
Director of Sponsor	Signature	Date

Protocol Signature Page

I agree to:

- Follow this study in strict accordance with the protocol, quality management practices of clinical drug trials and relevant laws and regulations.
- Keep all materials and information provided by Sinocelltech Ltd. in accordance with confidentiality requirements and indicate that they are confidential when submitted to Institution Review Committee or Independent Ethics Committee.

I have read the protocol in full and agree with all requirements.

____________________	____________________	____________________
Statistician	Signature	Date

Protocol Signature Page

I agree to:

- Conduct this study in strict accordance with the protocol, quality management practices of clinical drug trials and relevant laws and regulations.
- Keep all materials and information provided by Sinocelltech Ltd. in accordance with confidentiality requirements and indicate that they are confidential when submitted to Institution Review Committee or Independent Ethics Committee.

I have read the protocol in full and agree with all requirements.

______________________	______________________	______________________
Principal Investigator	Signature	Date

PROTOCOL SYNOPSIS

**Protocol No.**	SCTV01E-MRCT-2
**Protocol Title**	A randomized, double-blind, placebo-controlled Phase III clinical trial to evaluate the efficacy and safety of SCTV01E (A COVID-19 Alpha/Beta/Delta/Omicron Variants S-Trimer Vaccine) in healthy adults aged ≥18 years
**Version No.**	Version 6.0
**Version Date**	May 5, 2023
**Sponsor**	Sinocelltech Ltd.
**Study Phase**	Phase III
**Indication**	Prevention of COVID-19
**Target Population**	Individuals aged ≥18 years who previously received primary series COVID-19 vaccine or booster dose
**Study Objectives**	**Primary Objective:**<br>• To evaluate the protective efficacy of SCTV01E against symptomatic SARS-CoV-2 infection occurring 7 days after the study vaccine.<br>**Secondary Objective:**<br>• To evaluate the protective efficacy of SCTV01E against all infections (including asymptomatic infection), asymptomatic infection, obvious symptomatic infection of SARS-CoV-2, moderate and above, severe and above COVID-19 and death due to COVID-19 occurring 7 days after the study vaccine, respectively.<br>• To evaluate the protective efficacy of SCTV01E against all infections (including asymptomatic infection), asymptomatic infection, symptomatic infection and obvious symptomatic infection of

	SARS-CoV-2 occurring 14 days after the study vaccination, respectively.<br>• To evaluate the protective efficacy of SCTV01E against infection of SARS-CoV-2 variants and subvariants occurring from 14 days after the study vaccination.<br>• To evaluate the immunogenicity of SCTV01E.<br>• To evaluate the safety of SCTV01E.
**Study endpoint**	**<u>Primary endpoints</u>**<br>• Cases of the first occurrence of symptomatic infection of SARS-CoV-2 of any severity starting 7 days (≥8 days) post-vaccination.<br>**<u>Secondary endpoints:</u>**<br>***Efficacy***<br>• Cases of the first occurrence of all infection (including asymptomatic infection), asymptomatic infection, obvious symptomatic infection of SARS-CoV-2, moderate and above, severe and above COVID-19 and death due to COVID-19, respectively, starting 7 days (≥8 days) post-vaccination.<br>• Cases of the first occurrence of all infection (including asymptomatic infection), asymptomatic infection, symptomatic infection, and obvious symptomatic infection of SARS-CoV-2, respectively, starting 14 days (≥15 days) post-vaccination.<br>• Cases of the first occurrence of symptomatic infection, obvious symptomatic infection of SARS-CoV-2, moderate and above, severe and above COVID-19 and death due to COVID-19, respectively, caused by SARS-CoV-2 variants and subvariants starting 14 days (≥15 days) post-vaccination.<br>***Immunogenicity***<br>• Geometric mean titers (GMT) of neutralizing antibodies (nAb) against SARS-CoV-2 (including its variants and subvariants) on Days 7, 14, 28, 90, 180 and 365.<br>• Seroresponse of nAb (defined as a change from below the low limit

	of quantitation [LLOQ] to equal to or above LLOQ, or a ≥4-fold rise if the baseline is equal to or above LLOQ in nAb to SARS-CoV-2 variant from Day 0) rates on Days 7, 14, 28, 90, 180 and 365, respectively.<br>***Safety***<br>• Incidence and severity of solicited adverse events (AEs) of SCTV01E from day 0 (Day 0) to day 7 (Day 7).<br>• Incidence and severity of unsolicited AEs of SCTV01E from Day 0 to day 28 (Day 28).<br>• Incidence and severity of serious adverse events (SAEs) and adverse events of special interest (AESIs).
**Study Design**	SCT had initiated several clinical trials to evaluate the safety and immunogenicity of SCTV01E and SCTV01C, including one Phase III immunogenicity trial for SCTV01E and SCTV01C and three Phase I/II trials for SCTV01C. SCTV01E and SCTV01C were manufactured by the same platform, sharing the same process, similar molecular characteristics and clinical dosage, therefore, these trials provide sufficient supportive safety and immunogenicity clinical data prior to the start of this Phase III trial for SCTV01E. The details of these trials are summarized in the investigator's brochure.<br>The dose strength of SCTV01E is 30μg (5/5/5/15μg for TM22/TM23/TM28/TM41)/dose based on the nonclinical and clinical study of SCTV01E and SCTV01C.<br>The study is a randomized, double-blind, placebo-controlled Phase III study. It will evaluate the protective efficacy and safety of SCTV01E against COVID-19 in participants who were previously vaccinated with primary series of COVID-19 vaccines and /or received booster vaccination.<br>The study design is shown in Figure 1.<br>Approximately 10,000 participants who were previously vaccinated with the primary series of COVID-19 vaccine and/or received booster

vaccination and aged ≥ 18 years old will be enrolled. The exact number of participants enrolled in the study will depend on the infection rate of COVID-19 infection at the time of enrollment and the true vaccine efficacy of SCTV01E. If the attack rate is much higher, case accrual would be expected to be more rapid, enabling the study's primary endpoint to be evaluated much sooner, and the actual enrolled sample size may be less than planned.

A percentage of 5%~10% participants in the full analysis set will be set for both immunogenicity test and efficacy evaluation. (No limitation for the type of COVID-19 vaccine previously received or age ranges).

Participants will be randomly assigned to receive one dose of SCTV01E or placebo (normal saline) in a ratio of 1:1 on Day 0.

Participants are stratified by age (18-59 years, ≥60 years), the type of the last dose of COVID-19 vaccine previously received (inactivate, non-inactive) and region (study site).

Participants aged between 59 years to less than 60 years old will be taken as 59 years old;

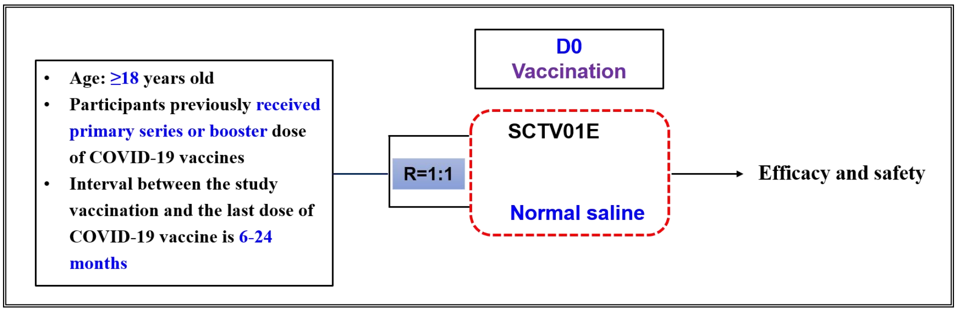

Figure 1 Study Design

Trial procedures:

The study consists of a screening period, a randomization and vaccination period, and a follow-up period.

Screening period: After participants sign the ICF, the screening phase visit will be conducted within 7 days before the study vaccination.

Randomization and vaccination period: The qualified participants will be randomized before the study vaccination. Randomized participants will be vaccinated on Day 0.

Follow-up period: The follow-up in this study includes efficacy

	follow-up, immunogenicity follow-up and safety follow-up. The follow-up period may be adjusted by the sponsor according to the pandemic of COVID-19 and the spreading variants of SARS-CoV-2.<br>**Efficacy follow-up:**<br>Follow-up of protective efficacy will be started after the study vaccination. All participants will be asked whether they have any signs/symptoms related to COVID-19 by phone call, short message, email or other contacting methods once a week. The frequency of the efficacy follow-up may be adjusted according to the process of the study. Meanwhile, the participants could spontaneously report any COVID-19-related symptoms at any time during the study period. SARS-CoV-2 RT-PCR test will be performed if participants have signs/symptoms related to COVID-19. If participants meet the criteria for symptomatic infection of SARS-CoV-2 for the primary study endpoint, the virus will be isolated from the nasal/nasopharyngeal/throat swab and viral sequencing will be performed to identify the SARS-CoV-2 variants.<br>Endpoint Adjudication Committee (EAC) will be set up in this phase III study to conduct independent evaluation and judgment for each case, to ensure a more accurate determination of the positive cases.<br>**Immunogenicity follow-up:** Participants in the immunogenicity subgroup will be sampled on Days 0 (before study vaccination), 14, and 28. 200 participants in the immunogenicity subgroup will be sampled on Days 7, 90, 180 and 365 for nAb of SARS-CoV-2 (including variants and subvariants).<br>**Safety follow-up:** All participants will be observed at site for at least 30 minutes after the study intervention. Both active monitoring and spontaneous reporting will be used. Solicited AEs within 7 days after the study vaccination and unsolicited AEs within 28 days after the study vaccination will be collected through vaccination record cards (VRCs).

	SAEs and AESIs will be actively monitored for about 365 days after the study vaccination.<br>**IDMC:** An Independent Data Monitoring Committee (IDMC) will be set up to review the safety data after the study vaccination,<br>Participants can choose whether to be blinded or not after obtaining statistically significant vaccine efficacy and adequate safety data. Participants will be encouraged to remain in the ongoing study until the end of the study, unless they request to withdraw from the study for any reason. SAEs and AESIs would be monitored during this time.<br>For participants who request to be unblinded and are confirmed to be placebo recipients, the following procedure is available:<br>1) remain in the study for continued follow-up;<br>2) withdraw from the study by vaccination with other marketed vaccines;<br>3) withdraw from the study for other reasons.<br>Participants who receive or request any other COVID-19 vaccination during the study should withdraw from the study.
**Total Number of Participants**	Approximately 10,000 participants are planned to be enrolled. The percentage of participants aged ≥60 years shouldn't be less than 20%, and the percentage of participants who have received 3 doses of inactive COVID-19 vaccines shouldn't be less than 60%. The exact number of participants enrolled in the study will depend on the infection rate of COVID-19 at the time of the enrollment and the true VE (vaccine efficacy) of SCTV01E. If the attack rate is much higher, case accrual would be expected to be more rapid, enabling the study's primary endpoint to be evaluated much sooner and the actual enrolled sample size may be less than planned.

**Study Site**	Global, multi-center.
**Study Duration**	The study duration will be approximately 365 days for each participant.
**Inclusion Criteria**	Participants are eligible to be included in the study only if the following conditions are met:<br>1. Male or female aged ≥18 years old when signing ICF (the specific age range depends on the authorized age range by the local regulatory authority);<br>2. Participants who were vaccinated with the primary series COVID-19 vaccine and/or received booster vaccination, and the interval between the last dose of COVID-19 vaccine and screening is 6-24 months;<br>3. The participant and/or his/her legal guardian and/or his entrusted person can sign written ICF, and can fully understand the trial procedure, the risk of participating in the trial, and other interventions that can be selected if they do not participate in the trial;<br>4. The baseline SARS-CoV-2 anti-Spike IgG will be measured at the screening period which should be lower than 338BAU/mL. It is aimed to recruit participants who are susceptible to SARS-CoV-2 infection (excluding the participants with baseline SARS-CoV-2 anti-Spike IgG ≥338BAU/mL, who are highly associated with recent infection or in a recovery stage from SARS-CoV-2 infection). Most importantly, the upper limit was based on data obtained from previous studies (phase I/II). However, we may make adjustments to the upper limit of IgG test results at screening depending on the real situation; (e.g., in regions with low incidence of SARS-CoV-2 infection, this criteria may be adjusted or not be applied, if the number of participants exceeding this threshold reaches a certain proportion, the participants will be restricted to enroll according to

	this upper limit, on the contrary, the participants will be sampled during screening period without waiting for the result before enrollment;<br>5. The participant and/or his/her legal guardian and/or his entrusted person can read, understand, and fill in record cards;<br>6. Healthy participants or participants with pre-existing medical conditions who are in stable condition. The "pre-existing medical conditions" include but are not limited to hypertension (systolic/diastolic pressure ≤ 155/100 mmHg), diabetes, Chronic cholecystitis and cholelithiasis, and chronic gastritis that meet the described criteria. A stable medical condition is defined as disease not requiring significant change in therapy or no need for hospitalization as a consequence of worsening disease state for at least 3 months prior to enrollment;<br>7. Fertile men and women of childbearing potential voluntarily agree to take effective contraceptive measures from signing ICF to 6 months after the study vaccination; the pregnancy test results of women of childbearing potential are negative on screening.
**Exclusion Criteria**	A participant who conforms to any of the following criteria should be excluded from the study:<br>1. A positive result of nucleic acid test or rapid antigen test for SARS-CoV-2 during the screening period;<br>2. Known history of SARS-CoV-2 infection (including asymptomatic and symptomatic infection of SARS-CoV-2) in the past 6 months before the study vaccination;<br>3. Presence of fever within 3 days before the study vaccination (≥37.3°C);<br>4. A history of allergic reactions to any vaccines or drugs, such as allergy, urticaria, severe skin eczema, dyspnea, laryngeal edema, and

	angioneurotic edema;<br>5. A medical or family history of seizure, epilepsy, encephalopathy and psychosis;<br>6. Immunocompromised patients suffering from immunodeficiency diseases, important organ diseases, immune diseases (including Guillain-Barre Syndrome [GBS], systemic lupus erythematosus, rheumatoid arthritis, ankylosing spondylitis, asplenia or splenectomy caused by any circumstances, and other immune diseases that may have an impact on immune response in the investigator's opinion), etc.;<br>7. Long-term use of immunosuppressant therapy or immunomodulatory drugs for >14 days within the six months prior to enrollment. Whereas short-term (≤14 days) use of oral, inhaled and topical steroids are allowed;<br>8. Patients on antituberculosis therapy;<br>9. Presence of severe or uncontrollable cardiovascular diseases, or severe or uncontrollable disorders related to the endocrine system, blood and lymphatic system, liver and kidney, respiratory system, metabolic and skeletal systems, or malignancies (skin basal cell carcinoma and carcinoma in-situ of the cervix are exceptions and will not be excluded), such as severe heart failure, severe pulmonary heart disease, unstable angina, liver failure, or uremia;<br>10. Contraindications for intramuscular injection or intravenous blood sampling, including thrombocytopenia and other blood coagulation disorders;<br>11. Participants who received any immunoglobulin or blood products in the previous 3 months before enrollment, or plan to receive similar products during the study;

	12. Participants who received other investigational drugs within 30 days before the study vaccination or who intend to participate in another clinical study at any time during the conduct of this study;<br>13. Participants who have acute illness, such as acute onset of chronic heart failure, acute sore throat, hypertensive encephalopathy, acute pneumonia, acute renal insufficiency, acute cholecystitis;<br>14. Participants vaccinated with influenza vaccine within 14 days or with other vaccines within 28 days before the study vaccination;<br>15. Those who donated blood or had blood loss (≥450 mL) within 3 months before the vaccination or plan to donate blood during the study period;<br>16. Those who are pregnant or breast-feeding or plan to be pregnant during the study period;<br>17. Those who plan to donate ovum or sperm during the study period;<br>18. Those who cannot follow the trial procedures, or cannot cooperate to complete the study due to planned relocation or long-term outings;<br>19. Those unsuitable for participating in the clinical trial as determined by the investigator because of other abnormalities that are likely to confuse the study results, or non-conformance with the maximal benefits of the participants;<br>20. Those who tested positive for HIV in terms of serology.
**Withdrawal Criteria**	**Withdraw by participant:**<br>The participants have the right to withdraw from the study in advance at any phase of the study; or they are lost to follow-up because they fail to receive follow-up visits according to the trial requirements although they have not specifically proposed to withdraw from the study.<br>The investigator should try to contact the participants who withdraw from the study in advance, record the causes for their early withdrawal in the

	original data, and inform the study team of the case.<br>**Withdrawal determined by the investigator:**<br>Withdrawal from the study means that the investigator may determine that the enrolled participant should withdraw from the study when he or she is not suitable for continuing the study during the trial, for the following reasons:<br>1) Participants developed intolerable AE, and the investigator determined that continuing the study would be detrimental to the participant's health;<br>2) Major protocol deviation that may affect the safety of the participant;<br>3) Other reasons that disqualify participants from continuing this study as determined by the investigator.<br>**Handling participants withdrawing:**<br>Investigators should try their best to get in touch with participants who have not been to the site for regular follow-up. Investigators will follow up with participants who withdraw from the study due to serious adverse events or adverse events until the adverse events disappear, ease, stabilize, or other outcomes. If a participant withdraws from the study, the nasal/nasopharyngeal/throat swab and blood samples collected before the withdrawal date can still be used for study analysis, unless the participant specifically requests otherwise.<br>The investigator should record relevant information about withdrawal from the study in the electronic case report form (eCRF), including whether the participant or the investigator made the withdrawal decision, and a detailed record of the specific circumstances:<br>1) Losing contact;<br>2) Death, the investigator should record the cause of death;

	3) For voluntary withdrawal, the investigator should record the withdrawal and reason;<br>• Participants voluntarily withdraw from all studies, including all study activities such as vaccination, biological sample collection and safety observation;<br>• Participants voluntarily withdraw from part of the study, such as only stopping vaccination or only stopping biological sample collection, etc., other studies specified in the plan should continue to be completed;<br>• Participants request to withdraw due to study-related reasons, such as intolerance of adverse events, etc.;<br>• Participants request to withdraw due to reasons unrelated to the study, such as long-term outings, relocation, etc.;<br>4) It is determined by investigators that the participant should withdraw from the study;<br>5) Other reasons.<br>If a participant withdraws from the study or terminates the study (including loss to follow-up) after enrollment, no participant replacement is allowed.<br>After unblinding, participants who still choose to stay in the study will continue their safety follow-up.
**Study Suspension/Termination Criteria**	**In one of the following situations, the trial should be suspended or terminated:**<br>• When the IDMC requires a suspension/complete termination of the trial and the sponsor agrees;<br>• When the sponsor requires a suspension/complete termination of the trial and gives reasons for it;<br>• When the Ethics Committee requires a suspension/complete termination of the trial and gives reasons for it;

	• When the regulatory agency requires a suspension/complete termination of the trial and gives reasons for it.
**Study Vaccine**	**Study Vaccine:** a COVID-19 Alpha/Beta/Delta/Omicron Variants S-Trimer Vaccine (SCTV01E);<br>Appearance: emulsified, white suspension (due to the presence of adjuvant);<br>Components:<br>• Main active ingredients: TM22 protein (Alpha Variant), TM23 protein (Beta Variant), TM28 protein (Delta Variant), TM41 protein (Omicron Variant);<br>• Adjuvant: SCT-VA02B;<br>• Excipients: citric acid monohydrate, trisodium citrate dihydrate, sodium chloride, polysorbate 80;<br>Dosage form: solution for injection;<br>Strength: 30μg [5/5/5/15μg for TM22 (Alpha) /TM23 (Beta) /TM28 (Delta) /TM41 (Omicron)]/0.5mL/vial;<br>Route of vaccination: intramuscular injection into the lateral deltoid of the upper arm;<br>Dosage of vaccination: 30μg;<br>Immunization procedure: single dose;<br>Storage conditions: stored and transported at 2 ~ 8℃ away from light;<br>Validity period: tentatively 24 months;<br>Manufacturer: Sinocelltech Ltd.<br>**Control:**<br>Normal saline will be used as placebo control in this study (0.5 mL/dose).
**Statistical Analysis**	**The main statistical analysis methods in this study are described in this chapter, and details will be listed in the statistical analysis plan.**<br>**Hypothesis:**

	Define VE (vaccine efficacy) as 1- hazard ratio (HR).<br>For the primary efficacy endpoint, the null hypothesis is<br>**H0**: The VE of SCTV01E versus control is ≤ 30%.<br>**H1**: The VE of SCTV01E versus control is > 30%.<br><br>**Sample size estimation:**<br>After the adjustments of epidemic prevention and control policy in Beijing, SCTV01E showed a VE of approximately 70%-77% in the real-world. A total of 59 symptomatic SARS-CoV-2 infection cases will provide approximately 90% power to detect a VE of 70% at one-sided type one error 0.025 among participants in the per-protocol set for efficacy (PPE).<br>The hypothesis of sample size for vaccine efficacy endpoint:<br>• The COVID-19 incidence rate per month in the control arm is about 1.5%<br>• The dropout rate during the study is about 10%<br>• The participants with positive results of PCR test at baseline or infection of SARS-CoV-2 within 7 days after the study vaccination should be excluded from the analysis of primary endpoint cases, which is about 15%<br>Based on the assumptions aforementioned, approximately 10,000 participants will be enrolled in this study. The final number of participants enrolled in the study will depend on the incidence of COVID-19 at the time of the enrollment and the accumulation of primary endpoint cases, and the actual enrolled sample size may vary accordingly. In addition, 5%-10% of participants will be involved in the immunogenicity subgroup.<br>**Statistical populations:**<br>**Full Analysis Set (FAS):** All randomized participants who received one

	dose of the investigational product (IP). Participants will be analyzed according to the treatment group to which they were randomized.<br>**Per-Protocol Set (PPS):** All participants in the FAS set who received one dose of IP per schedule and have no major protocol deviations, as determined and documented by the Sponsor prior to DBL and unblinding, that could impact critical or key study data. Participants will be analyzed according to the treatment group to which they were randomized.<br>**Per-Protocol Set for Efficacy (PPE):** Participants in the PPS set who have negative results of nucleic acid test and with total anti-spike antibodies < 338 BAU/mL at baseline, as well as no evidence for SARS-CoV-2 infection (positive results of antigen or nucleic acid test) within 7 days after the study vaccination. Participants in the PPE set are the main analysis population for VE assessment 7 days after the study vaccination.<br>**Per-Protocol Set for Efficacy-14 (PPE-14):** Participants in the PPS set who have negative results of nucleic acid test and with total anti-spike antibodies < 338 BAU/mL at baseline, as well as no evidence for SARS-CoV-2 infection (positive results of antigen or nucleic acid test) within 14 days after the study vaccination. Participants in the PPE-14 set are the main analysis population for VE assessment 14 days after the study vaccination.<br>**Safety Set (SS):** All randomized participants who received one dose of IP. The safety analysis will be based on the treatment group to which the participants receive. Individuals who received the study vaccination but were not randomized will be excluded from the safety set, the safety data of them will be listed separately.<br>**Immunogenicity full analysis set (I-FAS):** All participants in the FAS who had a valid immunogenicity test result prior to receiving IP and at

	least 1 valid result after receiving IP.<br>**Immunogenicity Per-Protocol Set (I-PPS):** All participants in the PPS who had a valid immunogenicity test result prior to receiving IP and at least 1 valid result after receiving IP.<br>Participants in the PPE set are the main analysis population for VE assessment (including primary endpoint) 7 days after the study vaccination. Participants in the PPE-14 set are the main analysis population for VE assessment 14 days after the study vaccination<br>Participants in the SS set are the main analysis population for safety.<br>Participants in the I-PPS set are the main analysis population for immunogenicity. Particularly, participants with evidence of SARS-CoV-2 infection prior to the target date of the analysis (e.g. 28 days after the study vaccination) will be excluded from the immunogenicity analysis.<br>**Statistical analysis methods:**<br>**<u>Efficacy analysis</u>**<br>Analysis of the primary endpoint will be performed based on PPE.<br>Primary endpoint: The vaccine efficacy will be estimated with 1–HR using a Cox proportional hazard regression model with separate baseline strata of age (18-59 years, ≥60 years) and the type of the last dose of COVID-19 vaccine previously received (inactive, non-inactive). Events that coincide in time will be processed based on the Efron method.<br>Subgroup analysis of the primary efficacy endpoint will be performed to assess the consistency of vaccine efficacy, such as different ages (18-59 years, ≥60 years), and the type of the last dose of COVID-19 vaccine previously received (inactive, non-inactive).<br>Details of the sensitivity analysis are described in section 11.4.4.<br>Similar statistical analysis model will be applied to VE assessment for both the primary endpoint and second endpoints.

	**<u>Immunogenicity Analysis</u>**<br>The Immunogenicity analysis will be based on I-PPS.<br>The GMT of neutralizing antibody for each group with corresponding 2-sided 95% CI will be estimated.<br>Comparisons between groups were based on age (18-59 years, ≥60 years) and the type of last dose of COVID-19 vaccines (inactive, non-inactive) as fixed effects ANOVA model<br>**<u>Safety Analysis</u>**<br>Safety analysis will be based on SS.<br>AEs and SAEs are encoded based on the *Medical Dictionary for Regulatory Activities* (MedDRA), and also based document the classified statistics were made according to the system organ class (SOC) and preferred term (PT). In this trial, the solicited adverse events (both systemic and local) will be summarized and classified according to PT.<br>In this trial, the treatment emergent adverse events (TEAEs) will be summarized, and the adverse medical conditions occurring before the study vaccination are listed. Unless otherwise specified, the adverse events as described below are TEAEs.<br>The number of occurrences, number of cases and incidence rate of AEs, SAEs and AESIs in each group will be summarized respectively. The adverse events related to the study vaccine, SAEs and AESIs will be listed.<br>Supplemental analysis will be performed on adverse events in the following populations:<br>• Participants who have negative results of PCR test at baseline and negative results of rapid antigen test on Day 7;<br>• Participants who have negative results of PCR test at baseline and negative results or absence of rapid antigen test on Day 7;

	• Participants who have positive results of PCR test at baseline.<br>**Multiplicity**<br>A fixed sequence hierarchical procedure will be followed to have the overall Type I error rate controlled at one-sided 0.025. The subsequent test will be performed as follows:<br>1) Primary endpoint: among the population in PPE, VE for symptomatic SARS-CoV-2 infection 7 days (≥8 days) after the study vaccination is higher than 30%;<br>2) Secondary endpoint: among the population in PPE, VE for obvious symptomatic SARS-CoV-2 infection 7 days (≥8 days) after the study vaccination is higher than 0%;<br>3) Secondary endpoint: among the population in PPE, VE for all infections of SARS-CoV-2 (including symptomatic and asymptomatic infection of SARS-CoV-2) 7 days (≥8 days) after the study vaccination is higher than 0%;<br>4) Secondary endpoint: among the population in PPE-14, VE for symptomatic infection of SARS-CoV-2 14 days (≥15 days) after the study vaccination is higher than 0%;<br>5) Secondary endpoint: among the population in PPE-14, VE for obvious symptomatic infection of SARS-CoV-2 14 days (≥15 days) after the study vaccination is higher than 0%;<br>6) Secondary endpoint: among the population in PPE, VE for asymptomatic infection of SARS-CoV-2 7 days (≥8 days) after the study vaccination is higher than 0%;<br>7) Secondary endpoint: among the population in PPE, VE for obvious symptomatic infection of SARS-CoV-2 7 days (≥8 days) after the study vaccination is higher than 30%;<br>8) Secondary endpoint: among the population in PPE, VE for all infections of SARS-CoV-2 7 days (≥8 days) after the study vaccination is higher than 30%;<br>9) Secondary endpoint: among the population in PPE-14, VE for symptomatic infection of SARS-CoV-2 14 days (≥15 days) after the study vaccination is higher than 30%;

	10) Secondary endpoint: among the population in PPE-14, VE for obvious symptomatic infection of SARS-CoV-2 14 days (≥15 days) after the study vaccination is higher than 30%.<br><br>**<u>Interim and final analysis</u>**<br>The is no planned interim analysis in this study. The final analysis will be performed for the primary endpoint at the significance level of 0.025 unilateral when more than 59 primary endpoint cases have been collected. The final analysis will be performed by an independent third party, the sponsor will set up an unblinded team for delivery. Investigators, participants and team members directly involved in this study will remain blinded for long-term efficacy and safety follow-up.
**Independent Data Monitoring Committee (IDMC)**	The sponsor will establish the IDMC to review the safety data of the clinical trials. The IDMC members include experts in the field of vaccine clinical trials, biostatisticians and epidemiologists. See the "IDMC charter" for details of its working documents.
**Endpoint Adjudication Committee (EAC)**	Endpoint Adjudication Committee (EAC) will be set up in this phase III study to conduct independent evaluation and judgment for each case, to ensure a more accurate determination of the positive cases.

Table 1 Schedule of Activities

	**Screening period**	**Vaccination**	**Follow-up period**					**Blinded or unblinded determined by the participant**	**Follow-up period**
**Visit**	**V1**	**V2**	**V3**	**V4**	**V5**	**V6&**	**V7&**	**V8▲**	**V9/EOS▲**
**Planned visit date**	**D-7～D0**	**D0**	**D7**	**D14**	**D28**	**D90**	**D180**	**After the final efficacy analysis**	**D365**
**Visit window period**	/	/	**+2 days**	**±3 days**	**+7 days**	**±10 days**	**±10 days**	/	**±10 days**
**Management and general procedure**									
Signing the informed consent form	●								
Assigning the screening number	●								
Confirm participant meets inclusion and exclusion criteria [1]	●	●★							
Demographic data [2]	●								
Recording the medical history [3]	●								
Nasal/pharyngeal/throat swab **rapid antigen test** for SARS-CoV-2 [4]	●		●	●					
Nasal/pharyngeal/throat swab **nucleic acid test** for SARS-CoV-2 [4]	●	●★	The sample will be collected from the participants who have COVID-19 related signs/symptoms, reference to Section 9.3.4						
Vital signs [5]	●	●★							
Physical examination [6]	●								
Anti-SARS-CoV-2 IgG antibodies [7]	●								
HIV testing (not applied in China)	●								
Urine pregnancy test (for women of childbearing potential only) [8]	●	●★							
Confirmation the selection of participants [9]								●	
Randomization		●*							
Vaccination		●							
**Immunogenicity follow-up**									
Neutralizing antibodies test [10]		●	●	●	●	●	●		●
**Efficacy follow-up visit**									
Efficacy follow-up		The follow-up visit will be conducted once a week by means of phone call, text message, e-mail or on site. On-site visit will only be conducted when investigators think it is necessary. The frequency of							

	Screening period	Vaccination	Follow-up period					Blinded or unblinded determined by the participant	Follow-up period
**Visit**	**V1**	**V2**	**V3**	**V4**	**V5**	**V6&**	**V7&**	**V8▲**	**V9/EOS▲**
**Planned visit date**	**D-7～D0**	**D0**	**D7**	**D14**	**D28**	**D90**	**D180**	**After the final efficacy analysis**	**D365**
**Visit window period**	/	/	**+2 days**	**±3 days**	**+7 days**	**±10 days**	**±10 days**	/	**±10 days**
		efficacy follow-up may be adjusted according to the process of the study.							
**Safety follow-up visit**									
Solicited AEs [11]		●							
Unsolicited AEs [11]		●							
SAEs and AESIs[12]		●	●	●	●	●#	●#	●#	●#
Observing for at least 30 minutes after the vaccination		●							
Distributing the vaccination record cards (VRCs)		●							
Reviewing and recovering the VRCs				●	●				
Distributing the thermometer and tape measure		●							
Recording the concomitant medication		●	●	●	●	●#	●#	●#	●#

Abbreviations: AE=Adverse Event, AESI=Adverse Event of Special Interest, SAE=Serious Adverse Event, VRC=Vaccination Record Card

Comments:

&: V6 and V7 are only applicable for participants in the immunogenicity subgroup.

▲: For V8 and V9, this visit will be done by phone call or other means of telecommunication, while it can also be done on-site if necessary, if the participants received other marketed COVID-19 vaccines, the participants should withdraw from the study.

★: If screening and vaccination are on the same day (D0), there is no need to repeat the items corresponding to '●★' before vaccination.

#: 28 days after each study vaccination, only the concomitant medication used to treat SAEs and AESIs should be recorded.

*: Participants are randomized after all the tests have been done and eligibility has been confirmed before the vaccination on the vaccination day.

1. Inclusion/exclusion criteria should be reviewed during the screening period and before vaccination on the day of vaccination.
2. Demographic data: including date of birth, sex, race/ethnicity, height, body weight, and BMI (derived from height and body weight). The participants should also provide contact information like their current phone number and/or E-mail. In subsequent follow-up visits, if the contact information is changed, it should be updated accordingly (if applicable).
3. Records of medical history: including the history of SARS-COV-2 vaccination, history of COVID-19, other vaccinations within 28 days, medication use within 28 days, major surgery, allergic history and other known significant diseases.
4. Nasal/pharyngeal/throat swab for rapid antigen test and nucleic acid test for SARS-CoV-2: A rapid antigen test or nucleic acid test should be performed at screening at the study site before vaccination. Participants with positive results will be excluded from the study. Nasal/pharyngeal/throat swab samples for nucleic acid test should be collected from all participants before vaccination and there is no need to wait for the report for vaccination. If screening vaccination is on the same day, there is no need for repeat nucleic acid tests. Before the participants signed the ICF, if the participants have been sampled for rapid antigen test or nucleic acid test during the screening period, there is no need for repeat test (determined by the investigators).
5. Vital signs: blood pressure, respiratory rate, pulse rate, and body temperature.
6. Physical examinations: general conditions, head & neck, lymph node, skin, chest, abdomen, musculoskeletal system and other examinations necessary for the study.
7. Anti-SARS-CoV-2 IgG antibodies: The baseline SARS-CoV-2 anti-Spike IgG will be measured during the screening period, participants will be excluded from the study if the baseline SARS-CoV-2 anti-Spike IgG is higher than the upper limit (in regions with low incidence of SARS-CoV-2 infection, this criteria may be adjusted or not be applied, if the number of participants exceeding this threshold reaches a certain proportion, the participants will be restricted to enroll according to this upper limit, on the contrary, the participants will be sampled during screening period without waiting for the result before enrollment). If the upper limit of baseline SARS-CoV-2 anti-Spike IgG >338BAU/mL was not the excluding criteria, the blood sample should be collected before the vaccination;
8. Urine pregnancy test (for women of childbearing potential only): the urine pregnancy test may be performed routinely, while the blood pregnancy test may be performed if the investigator deems it necessary. A woman is considered of childbearing potential (WOCBP), i.e. fertile, following menarche and until becoming post-menopausal unless permanently sterile. Permanent sterilization methods include hysterectomy, bilateral salpingectomy and bilateral oophorectomy. A postmenopausal state is defined as no menses for 12 months without an alternative medical cause.
9. For Participants who request to be unblinded and are confirmed to be placebo recipients, the following procedure is available: 1) remain in the study for continued follow-up; 2) withdraw from the study by vaccination with other marketed vaccines; 3) withdraw from the study for other reasons. All participants will be encouraged to remain in the

ongoing study until the end of the study, unless they request to withdraw from the study for any reason.

10. Neutralizing antibodies test: participants in the immunogenicity subgroup (5%-10% of the population in FAS) will be sampled on Days 0 (before vaccination), 14, 28. 200 participants in the immunogenicity subgroup will be sampled on Days 7, 90, 180 and 365 for nAb of SARS-CoV-2 (including variants and subvariants).
11. Solicited and unsolicited AEs: collect solicited AEs from D0 to D7, and unsolicited AEs from D0 to D28.

SAEs, and AESIs: SAEs, and AESIs will be collected on visit day or reported by participants actively at any time. If participants cannot come to the site on visit day, phone calls, short messages, email or other contacting methods will be used for safety follow-up.

A Randomized, Double-blind, Placebo-controlled Phase III Clinical Trial to Evaluate the Efficacy and Safety of SCTV01E （A COVID-19 Alpha/Beta/Delta/Omicron Variants S-Trimer Vaccine） in Healthy Adults Aged ≥ 18 Years

Sponsor: Sinocelltech Ltd.

Study facilities: Guizhou Center for Disease Control and Prevention

Sichuan Center for Disease Control and Prevention

Hunan Provincial Center for Disease Control and Prevention

Statistical Analysis Plan

Signature Page for Approval

Sponsor

Sponsor: Sinocelltech Ltd.

Statistics Director: [redacted]

Signature: Date:

Table of Contents

1. DESCRIPTION OF ABBREVIATIONS AND STATISTICS (IN ENGLISH) USED 1
2 STUDY OVERVIEW 2
2.1 INTRODUCTION 2
2.2 ANALYTICAL PURPOSE 2
2.3 CHANGES IN STATISTICAL ANALYSIS FROM THE PROTOCOL 2
3. STUDY OBJECTIVES 2
3.1 PRIMARY OBJECTIVE 2
3.2 SECONDARY OBJECTIVES 3
4. STUDY DESIGN 3
4.1 OVERALL DESIGN 3
4.2 RANDOMIZATION 4
4.3 SAMPLE SIZE 5
5. EVALUATION ENDPOINTS 5
5.1 EFFICACY ENDPOINTS 5
5.1.1 Primary efficacy endpoint 5
5.1.2 Secondary efficacy endpoints 5
5.2 SAFETY ENDPOINTS 7
6. ANALYSIS SETS 7
7. STATISTICAL ANALYSIS METHODS 8
7.1 GENERAL CONSIDERATIONS 8
7.1.1 General analytical methods 8
7.1.2 Related definitions and derivative rules 9
7.1.3 Analysis windows 10
7.1.4 Analysis software 10
7.1.5 Tables and listings 10
7.2 PARTICIPANT DISTRIBUTION 11
7.3 DEMOGRAPHIC DATA AND BASELINE CHARACTERISTICS 11
7.4 CONCOMITANT MEDICATIONS AND VACCINATIONS 12
7.5 STUDY HYPOTHESES 12
7.6 EFFICACY EVALUATION 12
7.6.1 Primary efficacy evaluation 12
7.6.2 Secondary efficacy evaluation 14
7.7 SAFETY EVALUATION 19
7.7.1 AEs 19
7.7.2 SAEs 20
7.7.3 AESIs 20
7.8 HANDLING OF MISSING DATA 20
7.9 SUBGROUP ANALYSIS 20
7.10 MULTIPLICITY 21
7.11 INTERIM ANALYSIS 22
REVISION HISTORY 23

1. Description of Abbreviations and Statistics (in English) Used

AE	Adverse Event
AESI	Adverse Event of Special Interest
COVID-19	Coronavirus Disease 2019
EAC	Endpoint Assessment/Adjudication Committee
FAS	Full Analysis Set
IDMC	Independent Data Monitoring Committee
I-FAS	Full Analysis Set for Immunogenicity
I-PPS	Per-Protocol Set for Immunogenicity
IPS	Immunogenicity Persistence Set
ITT	Intention to Treat
IWRS	Interactive Web Response System
LLOQ	Lower Limit Of Quantitation
LOCF	Last Observation Carried Forward
Max	Max
Mean	Mean
MedDRA	Medical Dictionary for Regulatory Activities
Median	Median
Min	Min
PPE	Per-Protocol Set for Efficacy
PPS	Per-Protocol Set
PCR	Polymerase Chain Reaction
PT	Preferred Term
SAE	Serious Adverse Event
SAP	Statistical Analysis Plan
SAS	Statistical Analysis System
SD	Standard Deviation
SOC	System Organ Class
SS	Safety Set
TEAE	Treatment Emergent Adverse Event
WHO DD	World Health Organization Drug Dictionary

2 Study Overview

2.1 Introduction

This document is the Statistical Analysis Plan (SAP) for "**A randomized, double-blind, placebo-controlled Phase III clinical trial to evaluate the efficacy and safety of SCTV01E (A COVID-19 Alpha/Beta/Delta/Omicron Variants S-Trimer Vaccine) in healthy adults aged ≥18 years** ", and mainly describes specific statistical analysis methods used to analyze and report baseline characteristics of participants and efficacy and safety evaluation data. The relevant statistical analysis results of this study may be used for the registration application of this product.

This SAP will be finalized and approved prior to database lock, and the corresponding statistical analysis programming will be refined as study data accumulate until database lock.

Sample statistical analysis tables for this SAP will be provided separately as attachments.

2.2 Analytical purpose

The purpose of this SAP is to describe specific statistical analysis methods used to analyze and report baseline characteristics of participants and efficacy and safety evaluation data. The corresponding statistical analysis results will be presented in the final clinical study report, and will also be used for registration application, article publication, and other clinical needs of this product.

Post-hoc exploratory analysis allows for further analysis of study data, but is not presented in this SAP due to unpredictability. Post-hoc exploratory analysis, if conducted subsequently, will be detailed in the final statistical analysis report or the final clinical study report.

Additional analysis for other purposes, such as article publication and requirements of regulatory authority or sponsor, is also not presented in this SAP due to unpredictability. The corresponding statistical analysis methods for additional analysis, if conducted subsequently, may not be detailed in the final clinical study report, but will be detailed in the document presenting the additional analysis results.

2.3 Changes in statistical analysis from the protocol

The statistical analysis planned in this SAP is consistent with those specified in the protocol.

3. Study Objectives

3.1 Primary objective

To evaluate the protective efficacy of SCTV01E against symptomatic SARS-CoV-2 infection occurring 7 days after the study vaccine.

3.2 Secondary objectives

- To evaluate the protective efficacy of SCTV01E against all infections (including asymptomatic infection), asymptomatic infection, obvious symptomatic infection of SARS-CoV-2, moderate and above, severe and above COVID-19 and death due to COVID-19 occurring 7 days after the study vaccine, respectively;
- To evaluate the protective efficacy of SCTV01E against all infections (including asymptomatic infection), asymptomatic infection, symptomatic infection and obvious symptomatic infection of SARS-CoV-2 occurring 14 days after the study vaccination, respectively;
- To evaluate the protective efficacy of SCTV01E against infection of SARS-CoV-2 variants and subvariants occurring from 14 days after the study vaccination;
- To evaluate the immunogenicity of SCTV01E;
- To evaluate the safety of SCTV01E.

4. Study Design

4.1 Overall design

This study is a randomized, double-blind, and placebo-controlled Phase III clinical trial to evaluate the protective efficacy and safety of SCTV01E against COVID-19 in participants who were previously vaccinated with primary series of COVID-19 vaccines and /or received booster vaccination. This study includes a screening period, a period for randomization and vaccination, and a follow-up period (including follow-up for protective efficacy, immunogenicity, and safety). About 10000 participants will be enrolled in the study, with 5%–10% of participants in the immunogenicity subgroup. A screening visit will be conducted within 7 days after all participants sign the ICF. The participants who meet the inclusion and exclusion criteria will be randomized to the SCTV01E group or the control group at a ratio of 1:1. Participants in the SCTV01E group will receive one dose of SCTV01E on Day 0; participants in the control group will receive one dose of normal saline on Day 0.

Protective efficacy evaluation: Follow-up for protective efficacy will begin after vaccination. Study staff will ask participants for signs/symptoms related to COVID-19 by phone call, short message (SMS), email, or other means of communication. The follow-up for protective efficacy may be adjusted according to the progress of the trial. Meanwhile, participants can spontaneously report any COVID-19-related symptoms at any time during the study. Two nasal/nasopharyngeal/oropharyngeal swabs will be collected from the participant if any of the relevant COVID-19 clinical symptoms is/are met. One will be used for antigen rapid test and/or

SARS-CoV-2 reverse-transcriptase–polymerase-chain-reaction (RT-PCR) test; the other will be used for virus sequencing to determine which SARS-CoV-2 variant is infected if the participant is diagnosed with COVID-19 with any symptoms that meet the primary study endpoints. In addition, rapid antigen tests for SARS-CoV-2 nasal/nasopharyngeal/oropharyngeal swabs will be performed on Days 7 and 14. In order to guarantee that more accurate judgments are made for the collected COVID-19 cases, an Endpoint Assessment or Adjudication Committee (EAC) will be set up in this Phase III clinical trial to uniformly conduct independent evaluation and judgment on primary indicators.

Immunogenicity evaluation: Blood samples will be collected on Day 0 (pre-vaccination), Day 14 and Day 28 from participants in the immunogenicity subgroup (approximately 5%–10% of randomized participants), of which 200 participants need to undergo additional scheduled sampling on Day 7, Day 90 and other longer periods (detailed in the visit flow chart of the protocol) for the detection of SARS-CoV-2 (including mutant strains and mutant subtype) neutralizing antibody.

Safety evaluation: All participants will be observed at the study site for at least 30 minutes after the study intervention. Information is collected through a combination of active surveillance and spontaneous reporting. Solicited adverse events (AEs) within 7 days after vaccination and unsolicited AEs within 28 days after vaccination will be collected via vaccination record cards (VRCs). Serious adverse events (SAEs) and adverse events of special interest (AESIs) will be collected through active surveillance until the participant completes (or withdraws early from) the study.

This study will be conducted by Guizhou Center for Disease Control and Prevention, Sichuan Center for Disease Control and Prevention, and Hunan Provincial Center for Disease Control and Prevention.

4.2 Randomization

This clinical trial is a randomized and double-blind Phase III clinical trial. Participants will be randomized to the study vaccine group or the placebo group at a ratio of 1:1. To maintain relative balance between groups, participants will be stratified and randomized by age (18–59 years, ≥ 60 years), vaccine type of the last previous dose (inactivated, non-inactivated), and region (study site). Blind codes for participant randomization and blind codes for drug numbers will be generated and maintained by a third party.

Participants eligible for screening will be randomized by the Interactive Web Response System (IWRS) and vaccinated according to random numbers. For participants who withdraw from the study for any reason after randomization, regardless of whether they are administrated with the study

vaccine or not, their random numbers will be retained, and they shall not participate in the study again.

4.3 Sample size

After the adjustment of Beijing epidemic prevention and control policy, the protective efficacy of SCTV01E is between 70% and 77% in the real-world study. Therefore, assuming a protective efficacy (VE) of 70% (Hazard Ratio = 0.3) for the PPE population (as defined in the Analysis Set section) 7 days post-vacciation, in the condition of 59 cases, there is approximately 90% power to reject the null hypothesis H0 at a one-sided Type I error level of 0.025. The following assumption will be considered for sample size estimation and approximately 10000 participants (adjusted for actual infection rates) will be included. The final number of participants enrolled depends on the actual infection rate and the cumulative number of COVID-19 events. Of these participants, 5%–10% will be included in the immunogenicity subgroup.

- In the control group, the symptomatic infection of SARS-CoV-2 within 1 month is about 1.5%.
- The dropout rate during the study is about 10%.
- Approximately 15% of participants could not be included in the assessment of the number of events after 7 days because they are PCR positive at baseline or have an infection within 0–7 days after vaccination.

5. Evaluation Endpoints

5.1 Efficacy endpoints

5.1.1 Primary efficacy endpoint

- **Cases of the first occurrence of symptomatic infection of SARS-CoV-2 of any severity starting 7 days (≥8 days) post-vaccination.**

5.1.2 Secondary efficacy endpoints

5.1.2.1 Protective efficacy endpoints

(1) Cases of first occurrence of obvious symptomatic infection of SARS-CoV-2 of any severity starting 7 days post vaccination;

(2) Cases of first occurrence of all infections of SARS-CoV-2 (including asymptomatic infection) starting 7 days post-vaccination;

(3) Cases of asymptomatic infection of SARS-CoV-2 infection starting 7 days post-vaccination;

(4) Cases of first occurrence of moderate and above COVID-19 events starting 7 days

post-vaccination;

(5) Cases of first occurrence of severe and above COVID-19 events starting 7 days post-vaccination;

(6) Cases of death due to SARS-CoV-2 infection starting 7 days post-vaccination;

(7) Cases of first occurrence of symptomatic infection of SARS-CoV-2 starting 14 days post vaccination (≥15 days);

(8) Cases of first occurrence of obvious symptomatic infection of SARS-CoV-2 starting 14 days post-vaccination (≥15 days);

(9) Cases of first occurrence of all infections of SARS-CoV-2 (including asymptomatic infection) starting 14 days post-vaccination (≥15 days);

(10) Cases of first occurrence of asymptomatic infection of SARS-CoV-2 starting 14 days post-vaccination (≥15 days);

(11) Cases of first occurrence of symptomatic infection of SARS-CoV-2 caused by SARS-CoV-2 variants and subvariants starting 14 days post-vaccination;

(12) Cases of first occurrence of obvious symptomatic infection of SARS-CoV-2 caused by SARS-CoV-2 variants and subvariants starting 14 days post-vaccination;

(13) Cases of first occurrence of moderate and above COVID-19 infection caused by SARS-CoV-2 variants and subvariants starting 14 days post-vaccination;

(14) Cases of first occurrence of severe and above COVID-19 infection caused by SARS-CoV-2 variants and subvariants starting 14 days post-vaccination;

(15) Cases of death caused by SARS-CoV-2 variants and subvariants starting 14 days post-vaccination.

5.1.2.2 Immunogenicity endpoints

5.1.2.2.1 Prime immunization

For participants in the immune subgroup, the following endpoints are defined for the assessment of neutralizing antibody against SARS-CoV-2 variants on Days 14 and 28 after vaccination.

(1) Geometric mean titer (GMT) and geometric mean increase (GMI);

(2) Neutralizing antibody seroresponse rate, where the seroresponse rate is defined as the proportion of participants whose antibody titer shifts from below LLOQ before study vaccination to ≥ LLOQ after vaccination, or from ≥ LLOQ before vaccination to ≥ 4-fold baseline after vaccination.

5.1.2.2.2 Persistence study

For participants in the immune subgroup participating in the immunogenicity persistence study, the following endpoints are similarly defined for immunogenicity assessment visits on Days 7, 14, 28, and 90 after vaccination and subsequent scheduled dates:

(1) GMT and GMI of neutralizing antibody against SARS-CoV-2;

(2) Serum positive rate of neutralizing antibody against SARS-CoV-2.

5.2 Safety endpoints

(1) AEs

Solicited AEs: Injection site (local) AEs and non-injection site (systematic) AEs actively collected in the solicited period (within 0–7 days after vaccination).

- **Local AEs:** Injection site pain, injection site tenderness, injection site erythema, injection site swelling, injection site induration, and injection site pruritus.
- **Systemic AEs:** Fever, nausea, vomiting, headache, fatigue, myalgia, arthralgia, chills, and diarrhea.

Unsolicited AEs: All AEs except solicited AEs.

(2) SAEs

(3) AESIs

6. Analysis Sets

(1) Full analysis set (FAS)

It includes all randomized participants who receive at least one dose of the investigational product (IP). Participants receiving a vaccine with an incorrect number are analyzed in the originally randomized treatment group according to the principle of intention to treat (ITT).

(2) Per-protocol set (PPS)

It includes participants in the FAS except those with major protocol violations judged to have a significant impact on the evaluation of protective efficacy on 7 days after vaccination. Exclusion criteria are determined and documented jointly by the sponsor and the investigator prior to DBL and unblinding.

(3) Per-protocol set for efficacy (PPE)

It includes the susceptible population in the PPS with negative PCR results at baseline (baseline IgG < 338 BAU/mL) with no evidence of infection within 7 days after vaccination. The PPE population is the primary analysis population for VE assessment 7 days post-vaccination.

(4) Per-protocol set for efficacy-14 (PPE-14)

It includes the susceptible population in the PPS with negative PCR results at baseline (baseline

IgG < 338 BAU/mL) with no evidence of infection within 14 days after vaccination. The PPE-14 population is the primary analysis population for VE assessment 14 days post-vaccination .

(5) Full analysis set for immunogenicity (I-FAS)

It includes the population with vaccination of the investigational product, pre-vaccination immunogenicity baseline data, and at least one post-vaccination evaluable immunogenicity data in all immunogenicity subgroups.

(6) Per-protocol set for immunogenicity (I-PPS)

It includes participants in the I-FAS with no major protocol violation that affects the immunogenicity assessment on Day 28 after vaccination. Particularly, for participants with evidence of SARS-CoV-2 infection, post-infection immunogenicity data will be excluded from analysis, and pre-infection immunogenicity data may still be included.

(7) Immunogenicity per-protocol evaluable (I-PPE)

It includes the susceptible population in the PPS with negative PCR results at baseline (baseline IgG < 338 BAU/mL). The I-PPE is the primary analysis population for immunogenicity. Particularly, for participants with evidence of SARS-CoV-2 infection, post-infection immunogenicity data will be excluded from analysis, and pre-infection immunogenicity data may still be included.

(8) Safety set (SS)

It includes all participants who receive one dose of the investigational product (IP). The participants receiving a vaccine with an incorrect number are analyzed in the group of the treatment that they actually receive. Participants who are not randomized but receive the study vaccine will not be included in the safety set, and their safety data will be presented separately.

The safety set is mainly used for safety evaluation of the vaccine.

The protective efficacy of the vaccine will also be assessed among the population in the per-protocol set with positive evidence before the exclusion analysis target date (such as 7/14 days) and other populations.

The above analysis sets will be determined by the sponsor, the principal investigator, statisticians and data manager together at the data review meeting.

7. Statistical Analysis Methods

7.1 General considerations

7.1.1 General analytical methods

- **Descriptive statistics**

Unless otherwise stated, the following descriptive statistical summaries are given by variable type:

- ✓ Continuous variables are summarized with the mean, standard deviation, median, minimum, and maximum.
- ✓ Categorical or ordinal variables are summarized with the frequency and percentage, where the denominator for percentage calculation is the number of participants with valid results in the corresponding analysis set.

➢ **Decimal places**

Unless otherwise specified, the decimal places in the analysis report are implemented according to the following rules:

- ✓ Consistent with the largest number of decimal places of the original data for the minimum and maximum;
- ✓ One more digit than the largest number of decimal places of the original data for the median, mean, and standard deviation;
- ✓ Two decimal places for the percentage, rate, and rate difference;
- ✓ Four decimal places if the P value is ≥ 0.0001; reported as "< 0.0001" if the P value is < 0.0001;
- ✓ Three decimal places for test statistics in all statistical tests;
- ✓ Two decimal places for derived data.

7.1.2 Related definitions and derivative rules

➢ **Baseline**

Unless otherwise stated, baseline is defined as the last non-missing value measured prior to vaccination.

➢ **Conversion of years, months and days**

Month = days / 30.4375, Year = days / 365.25, rounded to one decimal place.

➢ **Duration of study**

Taking the vaccination date as the study start date, the duration of study corresponding to the examination or event is calculated based on the study start date according to the following formula: Duration of study = Examination date - Study start date.

➢ **Adverse Events**

AE onset time (days) = Onset date of AE - Vaccination date.

Duration of AE (days) = End date of AE - Onset date of AE + 1.

➢ **Treatment emergent adverse events (TEAEs)**

TEAEs are defined as the adverse events that occur after vaccination (including the day of vaccination), and occur before vaccination but worsen after vaccination, and are programmed

according to the following rules:

- ✧ An AE that occurs after/on the vaccination date is counted as TEAE;
- ✧ An AE that occur before the vaccination date is counted as non-TEAE;
- ✧ If the onset time of an AE or the vaccination date is missing so that it is impossible to clearly determine whether the AE occurs after the first-dose vaccination, the AE is counted as TEAE.

The study is designed to statistically analyze the **TEAEs**, including AEs occurring within 28 days after vaccination. AEs that occur before vaccination and 28 days after vaccination are listed separately. Unless otherwise specified, the AEs described below are TEAEs.

- ➢ **Coding**

Adverse events and past medical history are coded according to the Chinese version of the *Medical Dictionary for Regulatory Activities* (MedDRA) V25.1. Previous and concomitant medications and previous and concomitant vaccinations are coded according to the Chinese version of the *World Health Organization Drug Dictionary* (WHO DD), 202209.

- ➢ **Previous and concomitant medications**

The following rules are adopted for the judgment of previous and concomitant medications:

- ✧ Medications with an end date prior to the vaccination date are counted as previous medications.
- ✧ Medications that start before/on and end after the vaccination date, or start after the vaccination date, are counted as concomitant medications.

- ➢ **Previous and concomitant vaccinations**

The judgment rules for previous and concomitant vaccinations are the same as those for previous/concomitant medications.

7.1.3 Analysis windows

For visits after the baseline, statistical analyses by visit are performed based on the scheduled visits, and the visits outside the protocol will not be considered. Test results of unscheduled visits in the protocol will be listed.

7.1.4 Analysis software

All statistical analyses are performed by the statistical software SAS 9.4 or above.

7.1.5 Tables and listings

- ➢ **Tables**

Data is generally summarized by groups (SCTV01E and placebo), and groups are generally displayed in columns.

- **Listings**

Unless otherwise specified, all listings include group and participant numbers, and priority is given to presenting the original data in SDTM. The information in listings is generally sequenced by group, participant number, visit time, or other relevant time (e.g., AE onset time).

7.2 Participant distribution

The number of participants that are screened, randomized, and complete the study, and the number of participants in each analysis set are summarized; the reasons for early withdrawal are analyzed. The participants failing the screening, the participants withdrawing early, and the participants not enrolled in the analysis sets are listed, respectively.

The incidence of protocol deviations/violations is calculated by total and classification, respectively, and a list of protocol deviations/violations will be provided.

7.3 Demographic data and baseline characteristics

Demographic data and baseline characteristics will be analyzed based on FAS, PPE, PPE-14, and I-PPE.

The descriptive statistics of the following demographic data and baseline characteristics will be given separately for participants in each group:

- Demographic data (including age, sex, height, weight, BMI, ethnicity, and race);
- Clinical characteristics during the screening period (including whether the participant has history of COVID-19 infection, the time from the last infection to this vaccination (months), whether the COVID-19 vaccine has been vaccinated before enrollment, the time from the last previous dose to this vaccination (months), the vaccine type of the last previous dose, the number of doses vaccinated, the types of each dose of vaccine, rapid antigen test results, nucleic acid test results, HIV test results, stratification of anti-SARS-CoV-2 IgG antibody level, and anti-SARS-CoV-2 IgG antibody results);
- Follow-up time of cases (including follow-up time of cases on Day 7 after vaccination, follow-up time of cases on Day 14 after vaccination, and follow-up time of cases after vaccination);
- Baseline vital signs (including systolic blood pressure, diastolic blood pressure, respiratory rate, pulse, body temperature);
- Baseline physical examination (including general examination, head and neck, lymph nodes, skin, chest, abdomen, musculoskeletal system, and others).

Time from the last previous infection to this vaccination (months) = (Vaccination date - Last infection date + 1) / 30.4375; the time from the last previous dose to this vaccination (months) =

(Vaccination date - Last dose date + 1) / 30.4375.

The previous medications and vaccinations are coded according to WHO DD (Chinese version, 202209), and statistical description is performed on the number of previous medications/vaccinations, number of participants involved, and frequency of medications/vaccinations by ATC2 classification and drug name, respectively.

The past medical history is coded according to MedDRA (V25.1_Chinese version), and statistical description is performed on the number of past medical history, number of participants involved, and frequency of past medical history by SOC and PT, respectively.

The demographic list, list of past medical history, list of COVID-19 infection history, list of COVID-19 vaccination history, physical examination list, list of vital signs, rapid antigen test list, nucleic acid test list, HIV test list, list of anti-SARS-CoV-2 IgG antibody, list of previous medications, and list of previous vaccinations are provided.

7.4 Concomitant medications and vaccinations

Concomitant medications and vaccinations will be analyzed based on SS.

The concomitant medications and vaccinations are coded according to WHO DD (Chinese version, 202209), and statistical description is performed on the number of concomitant medications/vaccinations, number of participants involved, and frequency of medications/vaccinations by ATC2 classification and drug name, respectively.

A list of concomitant medications and a list of concomitant vaccinations are provided.

7.5 Study hypotheses

The lower limit of the two-sided 95% confidence interval (i.e., one-sided 97.5% confidence interval) of the protective efficacy (VE) of the vaccine against first occurrence of symptomatic infection of SARS-CoV-2 after 7 days post vaccination of 1 dose of Recombinant S-Trimer Protein Subunit (Alpha/Beta/Delta/Omicron Variant) Vaccine for SARS-CoV-2 (SCTV01E, COVID-19 vaccine) versus 1 dose of normal saline in participants aged ≥ 18 years is > 30%. The following hypothesis tests are considered:

Null hypothesis H_0: VE ≤ 30%;

Alternative hypothesis H_1: VE > 30%.

7.6 Efficacy evaluation

7.6.1 Primary efficacy evaluation

✧ **Protective efficacy of the study vaccine against symptomatic infection of SARS-CoV-2 after 7 days post vaccination (≥8 days) versus placebo**

The Cox proportional hazards model is used to statistically analyze the protective efficacy of the study vaccine against symptomatic infection of SARS-CoV-2 after 7 days post-vaccination, with the person-years at risk as the dependent variable and with the age (18−59 years, ≥ 60 years), the vaccine type of the last previous dose (inactivated, non-inactivated), and the group as fixed effects. The protection rate of the vaccine and its 95% confidence interval are calculated according to the model. Events that coincide in time are handled based on the Efron method. If the model reaches convergence, the stratification factor of the vaccine type of the last previous dose will be removed from the model first; if the model still converges, the age stratification factor will be further removed.

Person-years at risk of the primary endpoints = (End/censored time of valid case surveillance period - Vaccination time - 7) / 365.25; where, the end/censored time of valid case surveillance period is calculated according to the following rules:

- For those who have symptoms and are diagnosed with COVID-19 before the data cut-off date, the end time is the start date of the first symptom (first onset date) of the target event or the date of the positive result for antigen or nucleic acid tests corresponding to the event, whichever comes earlier;
- For those with positive results of rapid antigen and/or laboratory RT-PCR tests post vaccination before the data cut-off date, but not diagnosed with the target event, the censored time is the earlier of the sampling dates corresponding to the positive results for antigen and RT-PCR tests;
- For those with negative results of rapid antigen and/or laboratory RT-PCR test post vaccination before the data cut-off date, but not diagnosed with the target event, the censored time is the last follow-up date of the case surveillance;
- For those vaccinated with other COVID-19 vaccines before the data cut-off date, the censored time is the date of first vaccination with other COVID-19 vaccines; those diagnosed with target events after vaccination with other COVID-19 vaccines are not included in the case count.

The Kaplan-Meier curve for incidence of symptomatic COVID-19 after Day 7 post vaccination is plotted.

The primary protective efficacy analysis will be based on PPE. In addition, the sensitivity analysis will be performed with the following methods for the primary protective efficacy:

Sensitivity analysis 1 (vaccine protection rate analysis based on exact Poisson regression model): By the exact Poisson regression model, statistical analysis of the protective efficacy of the study vaccine against symptomatic infection of SARS-CoV-2 starting 7 days post vaccination is

performed. The vaccine protection rate based on person-year incidence rate and its 95% confidence interval are estimated according to the model. Poisson regression model is constructed with the number of cases as the dependent variable, the age (18−59 years, ≥ 60 years), the vaccine type of the last previous dose (inactivated, non-inactivated), and the group as fixed effects, and person-years at risk as the offset, and the log link function is employed.

Sensitivity analysis 2 (vaccine protection rate analysis based on Cox model not including stratification factors): The Cox proportional hazards model is used to statistically analyze the protective efficacy of the study vaccine against symptomatic infection of SARS-CoV-2 starting 7 days post vaccination, with the person-years at risk as the dependent variable, and the group as the fixed effect. The protection rate of the vaccine and its 95% confidence interval are calculated according to the model.

7.6.2 Secondary efficacy evaluation

7.6.2.1 Secondary protective efficacy evaluation

(1) Protective efficacy of the study vaccine against first occurrence of all infections, obivious symptomatic infection of SARS-CoV-2, respectively, starting 7 days post vaccination (≥8 days) versus placebo

Statistical analysis is performed on the protective efficacy of the study vaccine against first occurrence of all infections and obvious symptomatic infection of SARS-CoV-2, respectively, 7 days post vaccination (≥8 days) versus placebo based on PPE using the same analytical methods as used for the primary efficacy evaluation.

Sensitivity analysis is performed on the protective efficacy of the study vaccine against first occurrence of all infections and obvious symptomatic infection of SARS-CoV-2, respectively, 7 days post vaccination (≥8 days) versus placebo based on PPE using the same sensitivity analysis method as used for the primary efficacy evaluation.

(2) Protective efficacy of the study vaccine against first occurrence of all infections of SARS-CoV-2 (including asymptomatic infection) starting 7 days post vaccination (≥8 days) versus placebo

Statistical analysis is performed on the protective efficacy of the study vaccine against all infectiosn of SARS-CoV-2 (including asymptomatic infection) occurring for the first time after 7 days post vaccination (≥8 days) versus placebo based on PPE using the same analytical methods as used for the primary efficacy evaluation.

Sensitivity analysis is performed on the protective efficacy of the study vaccine against all infections of SARS-CoV-2 (including asymptomatic infection) occurring after 7 days post vaccination (≥8 days) versus placebo based on PPE using the same sensitivity analysis method as used for the primary efficacy evaluation.

(3) Protective efficacy of the study vaccine against asymptomatic SARS-CoV-2 infection occurring for the first time after 7 days post vaccination (≥8 days) versus placebo

Statistical analysis is performed on the protective efficacy of the study vaccine against asymptomatic SARS-CoV-2 infection occurring for the first time after 7 days post vaccination (≥8 days) versus placebo based on PPE using the same analytical methods as used for the primary efficacy evaluation.

Sensitivity analysis is performed on the protective efficacy of the study vaccine against asymptomatic SARS-CoV-2 infection occurring for the first time after 7 days post vaccination (≥8 days) versus placebo based on PPE using the same sensitivity analysis method as used for the primary efficacy evaluation.

(4) Protective efficacy of the study vaccine against moderate and above COVID-19 with SARS-CoV-2 infection occurring for the first time after 7 days post vaccination (≥8 days) versus placebo

Statistical analysis is performed on the protective efficacy of the study vaccine against moderate and above COVID-19 occurring for the first time after 7 days post vaccination (≥8 days) versus placebo based on PPE using the same analytical methods as used for the primary efficacy evaluation.

(5) Protective efficacy of the study vaccine against severe and above COVID-19 occurring for the first time after 7 days post vaccination (≥8 days) versus placebo

Statistical analysis is performed on the protective efficacy of the study vaccine against severe and above COVID-19 occurring for the first time after 7 days post vaccination (≥8 days) versus placebo based on PPE using the same analytical methods as used for the primary efficacy evaluation.

(6) Protective efficacy of the study vaccine against COVID-19-induced death occurring for the first time after 7 days post vaccination (≥8 days) versus placebo

Statistical analysis is performed on the protective efficacy of the study vaccine against COVID-19-induced death occurring for the first time after 7 days post vaccination (≥8 days) versus placebo based on PPE using the same analytical methods as used for the primary efficacy evaluation.

(7) Protective efficacy of the study vaccine against symptomatic infection of SARS-CoV-2 after 14 days post vaccination (≥15 days) **versus placebo**

Statistical analysis is performed on the protective efficacy of the study vaccine against symptomatic infection of SARS-CoV-2 after 14 days post vaccination (≥15 days) versus placebo based on PPE-14 using the same analytical methods as used for the primary efficacy evaluation.

Sensitivity analysis is performed on the protective efficacy of the study vaccine against symptomatic infection of SARS-CoV-2 after 14 days post vaccination (≥15 days) versus placebo based on PPE-14 using the same sensitivity analysis method as used for the primary efficacy evaluation.

(8) Protective efficacy of the study vaccine against obvious symptomatic infection of SARS-CoV-2 after 14 days post vaccination (≥15 days) versus placebo

Statistical analysis is performed on the protective efficacy of the study vaccine against obvious symptomatic infection of SARS-CoV-2 for the first time after 14 days post vaccination (≥15 days) versus placebo based on PPE-14 using the same analytical methods as used for the primary efficacy evaluation.

Sensitivity analysis is performed on the protective efficacy of the study vaccine against obvious symptomatic infection of SARS-CoV-2 for the first time after 14 days post vaccination (≥15 days) versus placebo based on PPE-14 using the same sensitivity analysis method as used for the primary efficacy evaluation.

(9) Protective efficacy of the study vaccine against all infections of SARS-CoV-2 (including asymptomatic infection) occurring for the first time after 14 days post vaccination (≥15 days) versus placebo

Statistical analysis is performed on the protective efficacy of the study vaccine against all infections of SARS-CoV-2 (including asymptomatic infection) occurring for the first time after 14 days post vaccination (≥15 days) versus placebo based on PPE-14 using the same analytical methods as used for the primary efficacy evaluation.

Sensitivity analysis is performed on the protective efficacy of the study vaccine against all infections of SARS-CoV-2 (including asymptomatic infection) occurring after 14 days post vaccination (≥15 days) versus placebo based on PPE-14 using the same sensitivity analysis method as used for the primary efficacy evaluation.

(10) Protective efficacy of the study vaccine against asymptomatic SARS-CoV-2 infection occurring for the first time after 14 days post vaccination (≥15 days) versus placebo

Statistical analysis is performed on the protective efficacy of the study vaccine against asymptomatic SARS-CoV-2 infection occurring for the first time after 14 days post vaccination (≥15

days) versus placebo based on PPE-14 using the same analytical methods as used for the primary efficacy evaluation.

Sensitivity analysis is performed on the protective efficacy of the study vaccine against asymptomatic SARS-CoV-2 infection occurring for the first time after 14 days post vaccination (≥15 days) versus placebo based on PPE-14 using the same sensitivity analysis method as used for the primary efficacy evaluation.

(11) Protective efficacy of the study vaccine against symptomatic infection of SARS-CoV-2 caused by SARS-CoV-2 variants and subvariants occurring for the first time after 14 days post vaccination (≥15 days) versus placebo

Statistical analysis is performed on the protective efficacy of the study vaccine against symptomatic infection of SARS-CoV-2 caused by SARS-CoV-2 variants and subvariants occurring for the first time after 14 days post vaccination (≥15 days) versus placebo based on PPE-14 using the same analytical methods as used for the primary efficacy evaluation.

(12) Protective efficacy of the study vaccine against obvious symptomatic infection of SARS-CoV-2 caused by SARS-CoV-2 variants and subvariants occurring for the first time after 14 days post vaccination (≥15 days) versus placebo

Statistical analysis is performed on the protective efficacy of the study vaccine against obvious symptomatic infection of SARS-CoV-2 caused by SARS-CoV-2 variants and subvariants occurring for the first time after 14 days post vaccination (≥15 days) versus placebo based on PPE-14 using the same analytical methods as used for the primary efficacy evaluation.

(13) Protective efficacy of the study vaccine against moderate and above COVID-19 caused by SARS-CoV-2 variants and subvariants occurring for the first time after 14 days post vaccination (≥15 days) versus placebo

Statistical analysis is performed on the protective efficacy of the study vaccine against moderate and above COVID-19 caused by SARS-CoV-2 variants and subvariants occurring for the first time after 14 days post vaccination (≥15 days) versus placebo based on PPE-14 using the same analytical methods as used for the primary efficacy evaluation.

(14) Protective efficacy of the study vaccine against severe and above COVID-19 caused by SARS-CoV-2 variants and subvariants occurring for the first time after 14 days post vaccination (≥15 days) versus placebo

Statistical analysis is performed on the protective efficacy of the study vaccine against severe and above COVID-19 caused by SARS-CoV-2 variants and subvariants occurring for the first time

after 14 days post vaccination (≥15 days) versus placebo based on PPE-14 using the same analytical methods as used for the primary efficacy evaluation.

(15) Protective efficacy of the study vaccine against COVID-19-induced death caused by SARS-CoV-2 variants and subvariants occurring for the first time after 14 days post vaccination (≥15 days) versus placebo

Statistical analysis is performed on the protective efficacy of the study vaccine against COVID-19-induced death caused by SARS-CoV-2 variants and subvariants occurring for the first time after 14 days post vaccination (≥15 days) versus placebo based on PPE-14 using the same analytical methods as used for the primary efficacy evaluation.

7.6.2.2 Immunogenicity

7.6.2.2.1 Prime immunization

The main results of the immunogenicity analysis for the prime immunization will be based on the I-PPE population. The I-PPS will also be analyzed. Particularly, post-infection immunogenicity data for SARS-CoV-2 infection will be excluded from analysis, and pre-infection data may still be included. Exploratory analysis will also be performed on participants who are infected at baseline (positive for nucleic acid or antigen tests).

The GMT of neutralizing antibody against SARS-CoV-2 (including variants or mutant subtypes) in the immunogenicity subgroup at each time point post vaccination is analyzed by the covariance model. The model is constructed with antibody GMT post vaccination after logarithmic transformation at each time point as the dependent variable, antibody GMT before vaccination after logarithmic transformation at each time point as the covariate, the age (18–59 years, ≥ 60 years), the vaccine type of the last previous dose (inactivated, non-inactivated), and the group as fixed effects. The arithmetic mean of antibody post vaccination after logarithmic transformation at each time point in each group and the least squares mean (LSM) of the difference between groups are calculated according to the model; after inverse logarithmic transformation, the LSM and 95% confidence interval of antibody GMT and GMT ratio (test group / control group) at each time point post vaccination in each group are calculated.

The geometric mean and two-sided 95% confidence interval are used to statistically describe anti-SARS-CoV-2 neutralizing antibody GMT and GMI at each time point in the test group and the control group under the immunogenicity subgroup, respectively.

The seroresponse rates of anti-SARS-CoV-2 neutralizing antibody in the test group and control group at each time point post vaccination are calculated, respectively, and their 95% confidence

intervals are calculated by Clopper-Pearson method. The ratio difference (test group - control group) and its two-sided 95% confidence interval are calculated using the CMH- χ^2 test including randomization stratification factors, and the difference between groups is statistically tested.

The histogram of anti-SARS-CoV-2 neutralizing antibody titer in the test group and the control group under the immunogenicity subgroup in the prime immunization, the forest plot of anti-SARS-CoV-2 neutralizing antibody GMT in each subgroup, and the forest plot of anti-SARS-CoV-2 neutralizing antibody seroresponse rate in each subgroup are drawn, respectively.

7.6.2.2.2 Persistence study

The immunogenicity analysis for the persistence will be based on subgroups of I-PPE that participate in the immune persistence study. The visits to be analyzed include scheduled visits on Days 7, 14, 28, 90, and other dates post vaccination until the end of the study.

GMT and two-sided 95% confidence interval are used to statistically describe anti-SARS-CoV-2 neutralizing antibody in the test group and the control group under the immunogenicity subgroup, respectively. An ANCOVA model similar to that used for the prime immunization will be used for comparison between groups.

The serum positive rates of anti-SARS-CoV-2 neutralizing antibody in the test group and control group at each time point for the persistence study are calculated, respectively, and their 95% confidence intervals are calculated by Clopper-Pearson method. Differences between groups are statistically tested using CMH-χ^2 test including randomization stratification factors.

The trend chart of anti-SARS-CoV-2 neutralizing antibody titer over time in the test group and control group during the persistence study are plotted, respectively.

7.7 Safety evaluation

7.7.1 AEs

AEs are coded using MedDRA (V25.1_Chinese version), and classified and counted according to the System Organ Class (SOC) and Preferred Term (PT). In addition, solicited AEs will be categorized by local AEs and systemic AEs specified in the protocol.

The number of participants and incidence of AEs in each group are calculated as follows:

- All AEs;
- AEs related to the study vaccine;
- AEs with an incidence of ≥ 0.1% in any group;
- AEs related to the study vaccine with an incidence of ≥ 0.1% in any group;
- AEs with an incidence of ≥ 1% in any group;

- AEs related to the study vaccine with an incidence of ≥ 1% in any group;
- AEs with an incidence of ≥ 10% in any group;
- AEs related to the study vaccine with an incidence of ≥ 10% in any group;
- AEs of different severity (Grade 1, Grade 2, Grade 3, Grade 4, Grade 5, ≥ Grade 2, and ≥ Grade 3);
- AEs related to the study vaccine of different severity;
- AEs by occurring time (within 30 min, 0–7 days, 0–14 days, 8–28 days);
- AEs related to the study vaccine with different onset times;
- AEs leading to withdrawal;
- AEs related to the study vaccine leading to withdrawal.

The severity and the relatedness to the study drug of the AEs are described statistically.

A list of adverse events is provided.

7.7.2 SAEs

The number of participants and incidence of SAEs in each group are calculated as follows:

- All SAEs;
- SAEs related to the study vaccine;
- SAEs related to the study vaccine with severity ≥ Grade 3;

A list of SAEs is provided.

7.7.3 AESIs

The number of participants and incidence of AESIs in each group are calculated as follows:

- All AESIs;
- AESIs related to the study vaccine;

A list of AESIs is provided.

7.8 Handling of missing data

In the evaluation of protective efficacy post vaccination, if the participant misses a follow-up, he/she will be considered non-infected for that follow-up to evaluate the protective efficacy.

In the study, the missing data of the safety evaluation endpoints and immunogenicity endpoints are not processed.

7.9 Subgroup analysis

If data permit, the following subgroups will be analyzed separately for protective efficacy and

immunogenicity:

- Age (18–59 years, ≥ 60 years)
- Sex (male, female)
- Time since last previous vaccination (6–12 months, 13–24 months)
- Vaccine type of the last previous dose (inactivated, non-inactivated)

In particular, if the model does not converge due to too few observed events during subgroup analysis, the factor of groups is only retained in the subgroup analysis model.

7.10 Multiplicity

The overall Type I error of the study will be controlled to a one-sided 0.025 using a hierarchical test in a fixed sequence as follows:

(1) Primary endpoint: VE > 30% against symptomatic infection of SARS-CoV-2 after 7 days post vaccination (≥8 days) in PPE population;

(2) Secondary endpoint: VE > 0% against obvious symptomatic infection of SARS-CoV-2 after 7 days post vaccination (≥8 days) in PPE population;

(3) Secondary endpoint: VE > 0% against all infections of SARS-CoV-2 after 7 days post vaccination (≥8 days) in PPE population;

(4) Secondary endpoint: VE > 0% against symptomatic infection of SARS-CoV-2 after 14 days post vaccination (≥15 days) in PPE-14 population;

(5) Secondary endpoint: VE > 0% against obvious symptomatic infection of SARS-CoV-2 after 14 days post vaccination (≥15 days) in PPE-14 population;

(6) Secondary endpoint: VE > 0% against asymptomatic SARS-CoV-2 infection after 7 days post vaccination (≥8 days) in PPE population;

(7) Secondary endpoint: VE > 30% against obvious symptomatic infection of SARS-CoV-2 after 7 days post vaccination (≥8 days) in PPE population;

(8) Secondary endpoint: VE > 30% against all infections of SARS-CoV-2 after 7 days post vaccination (≥8 days) in PPE population;

(9) Secondary endpoint: VE > 30% against symptomatic infection of SARS-CoV-2 after 14 days post vaccination (≥15 days) in PPE-14 population;

(10) Secondary endpoint: VE > 30% against obvious symptomatic infection of SARS-CoV-2 after 14 days post vaccination (≥15 days) in PPE-14 population.

In the other endpoints of efficacy, immunogenicity and safety, the calculated P value is just a nominal P value; it is mainly used to describe association strength between the endpoints and treatment subgroups, and is not used as the basis for formal statistical inference.

7.11 Interim analysis

There will be no planned interim analysis plan for this study. The study will conduct a final analysis of the primary endpoint at a one-sided significance level of 0.025 when at least 59 target events for the primary endpoint have accumulated. This analysis will be performed by an independent third party and the sponsor will establish an unblinded submission team to complete the submission. Investigators, participants, and team members directly involved in the project will remain blinded for long-term protective efficacy and safety follow-up.

REVISION HISTORY

Version	Version Date	Prepared by	Revision
V1.0	05/05/2023	Liu Dongfang	Initial Version
