# Supplementary material for: Efficacy of the tetravalent protein COVID-19 vaccine, SCTV01E: a phase 3 double-blind, randomized, placebo-controlled trial
Source: Nat Commun. 2024 Jul 24;15:6255. doi: 10.1038/s41467-024-49832-7 (PMC11269576; doi:10.1038/s41467-024-49832-7)
Supplement: Supplementary file 3 — Reporting Summary [file 41467_2024_49832_MOESM3_ESM.pdf]

# nature portfolio

Corresponding author(s): Liangzhi Xie

Last updated by author(s): Jun 3, 2024

# Reporting Summary

Nature Portfolio wishes to improve the reproducibility of the work that we publish. This form provides structure for consistency and transparency in reporting. For further information on Nature Portfolio policies, see our Editorial Policies and the Editorial Policy Checklist.

## Statistics

For all statistical analyses, confirm that the following items are present in the figure legend, table legend, main text, or Methods section.

| n/a | Confirmed | |
|---|---|---|
| ☐ | ☒ | The exact sample size ($n$) for each experimental group/condition, given as a discrete number and unit of measurement |
| ☐ | ☒ | A statement on whether measurements were taken from distinct samples or whether the same sample was measured repeatedly |
| ☐ | ☒ | The statistical test(s) used AND whether they are one- or two-sided<br>*Only common tests should be described solely by name; describe more complex techniques in the Methods section.* |
| ☐ | ☒ | A description of all covariates tested |
| ☐ | ☒ | A description of any assumptions or corrections, such as tests of normality and adjustment for multiple comparisons |
| ☐ | ☒ | A full description of the statistical parameters including central tendency (e.g. means) or other basic estimates (e.g. regression coefficient) AND variation (e.g. standard deviation) or associated estimates of uncertainty (e.g. confidence intervals) |
| ☐ | ☒ | For null hypothesis testing, the test statistic (e.g. $F$, $t$, $r$) with confidence intervals, effect sizes, degrees of freedom and $P$ value noted<br>*Give P values as exact values whenever suitable.* |
| ☒ | ☐ | For Bayesian analysis, information on the choice of priors and Markov chain Monte Carlo settings |
| ☒ | ☐ | For hierarchical and complex designs, identification of the appropriate level for tests and full reporting of outcomes |
| ☐ | ☒ | Estimates of effect sizes (e.g. Cohen's $d$, Pearson's $r$), indicating how they were calculated |

*Our web collection on statistics for biologists contains articles on many of the points above.*

## Software and code

Policy information about availability of computer code

| | |
|---|---|
| Data collection | The electronic data capture system (EDC) is used for data collection and no custom codes have used in the study. |
| Data analysis | SAS software (version 9.4). |

For manuscripts utilizing custom algorithms or software that are central to the research but not yet described in published literature, software must be made available to editors and reviewers. We strongly encourage code deposition in a community repository (e.g. GitHub). See the Nature Portfolio guidelines for submitting code & software for further information.

## Data

Policy information about availability of data

All manuscripts must include a data availability statement. This statement should provide the following information, where applicable:

- Accession codes, unique identifiers, or web links for publicly available datasets
- A description of any restrictions on data availability
- For clinical datasets or third party data, please ensure that the statement adheres to our policy

Data associated with this study are provided in the paper or supplementary materials. Source data for Figs. 4 and 5 and the protocol and statistical analysis plan are provided as Supplementary information. As the trial is still ongoing, anonymized participant data will be made available when the trials are complete, upon requests directed to the corresponding author. Proposals will be reviewed and approved by the sponsor, investigator, and collaborators based on the scientific merit of the request. After approval of a proposal, data can be shared through a secure online platform after signing a data access agreement.

## Research involving human participants, their data, or biological material

Policy information about studies with human participants or human data. See also policy information about sex, gender (identity/presentation), and sexual orientation and race, ethnicity and racism.

| | |
|---|---|
| Reporting on sex and gender | Regarding sex and gender, the study considered sex in its design and relied on self-reported information from participants to determine their sex. |
| Reporting on race, ethnicity, or other socially relevant groupings | This study does not involve the use of socially constructed or socially relevant categorization variables. |
| Population characteristics | Between December 26, 2022 and January 15, 2023, a total of 11,010 participants were initially screened for eligibility. Of them, 9,223 individuals were randomized at a 1:1 ratio to receive either SCTV01E or placebo (Figure 1). The Full Analysis Set (FAS) included 9,196 participants, with 4,595 in the placebo group receiving one dose of normal saline and 4,601 in the SCTV01E group receiving one dose of SCTV01E. Among the FAS population, 6,944 (75.5%) participants were between the ages of 18 and 59 years, while 2,252 (24.5%) were 60 years and older. 5,274 (57.4%) of participants were male. The demographic characteristics of the participants were well-balanced in the two groups (Table S1). The mean (SD) body mass index (BMI) was 24.1 (3.5) in the placebo group, and 24.2 (3.5) in the SCTV01E group, respectively. Most participants in both groups tested negative for SARS-CoV-2 nucleic acid at baseline, with 3877 (84.4%) in the placebo group and 3961 (86.1%) in the SCTV01E group, while the results were missing for a small number of participants (3 in the placebo group and 6 in the SCTV01E group). The baseline total anti-spike IgG levels were measured in both the placebo group and the SCTV01E group. In the placebo group, the mean (SD) baseline total anti-spike IgG was 1690.7 (1421.5) BAU/mL, while in the SCTV01E group, it was 1693.7 (1420.3) BAU/mL. A total of 1638 participants (35.6%) in the placebo group and 1650 participants (35.9%) in the SCTV01E group had a baseline total anti-spike IgG level below 338 BAU/mL (The 338 BAU/mL IgG level was selected as an indicator of recent infection.). 3406 (74.1%) participants in the placebo group and 3400 (73.9%) participants in the SCTV01E group had received an inactivated COVID-19 vaccine. The mean (SD) time since last vaccination were 13.2 (2.95) months in the placebo group, 13.1 (2.98) in the vaccine group, respectively. 967 (21.0%) participants in the placebo group and 1017 (22.1%) in the SCTV01E group had a pre-existing comorbidity. The Per-Protocol Efficacy (PPE) set comprised participants who had negative results on both the nucleic acid test and rapid antigen test during screening, and whose baseline levels of anti-spike IgG were less than 338 BAU/mL. At the cutoff date, a total of 1309 participants in the placebo group and 1314 participants in the SCTV01E group were included in the PPE analysis. The demographic characteristics of PPE (Table S2) were also well-balanced in the two groups. All participants received COVID-19 vaccines based on the ancestral strain before enrollment, and their prior vaccination is provided in Table S3. |
| Recruitment | This phase 3 study was a randomized, double-blind, placebo-controlled evaluation of the efficacy and safety of SCTV01E, a tetravalent SARS-CoV-2 trimeric spike protein vaccine, in adults aged 18 years and above. Participants were recruited from 9 study sites located across three provinces in China, including Sichuan, Guizhou, and Hunan.<br><br>Eligible participants were adults aged 18 years or above who had previously received the primary series of COVID-19 vaccine or booster vaccination, with a 6 to 24-month vaccination interval between the last dose COVID-19 vaccine and the study vaccination. Participants were excluded if they had a fever (temperature ≥37.3?) within three days before the study vaccination, a history of SARS-CoV-2 infection within 6 months, or a positive result for nasal/nasopharyngeal/throat swab nucleic acid test or rapid antigen test during screening. Full details related to the inclusion and exclusion criteria are provided in the trial protocol. |
| Ethics oversight | The protocol of this study, the written informed consent form, and other information related to participants were approved by the clinical research ethics board of the Sichuan/Guizhou/Human Provincial Center for Disease Control and Prevention (China). This trial followed the Declaration of Helsinki, Good Clinical Practice (GCP) requirements, and related regulations issued by authorities. |

Note that full information on the approval of the study protocol must also be provided in the manuscript.

# Field-specific reporting

Please select the one below that is the best fit for your research. If you are not sure, read the appropriate sections before making your selection.

☒ Life sciences ☐ Behavioural & social sciences ☐ Ecological, evolutionary & environmental sciences

For a reference copy of the document with all sections, see nature.com/documents/nr-reporting-summary-flat.pdf

# Life sciences study design

All studies must disclose on these points even when the disclosure is negative.

| | |
|---|---|
| Sample size | For the primary endpoint analysis, the efficacy will be demonstrated if the null hypothesis is rejected when the lower limit of the 2-sided 95% CI of the VE is greater than 30%. A total of 59 symptomatic SARS-CoV-2 infection cases will provide approximately 90% power to detect a VE of 70% at one-sided type one error 0.025. The study is case-driven, approximately 10,000 participants were enrolled in the study to cumulate the target number of symptomatic SARS-CoV-2 infection cases. |
| Data exclusions | All available safety and immunogenicity data were included. |

| | |
|---|---|
| Replication | The immunogenicity assays were performed once and each sample were tested duplicated. |
| Randomization | Interactive Network Response System (IWRS) was used to randomize the eligible participants prior to study vaccination, they were stratified by age (aged 18-59 years, ≥60 years), and the type of COVID-19 vaccine last received (inactivated vaccine, non-inactivated vaccine). The randomization codes were generated via block randomization using SAS software (Version 9.4). |
| Blinding | This study was conducted as a double-blind trial. Everyone involved, including participants, investigators, clinical research associates, data analysts, and laboratory staff, remained unaware of group assignments. Non-blind teams handled tasks such as vaccine reception, management, allocation, injection, packaging recovery, and overall drug management. They were strictly prohibited from participating in any study-related assessments or contacting participants for data collection after vaccine administration. |

# Reporting for specific materials, systems and methods

We require information from authors about some types of materials, experimental systems and methods used in many studies. Here, indicate whether each material, system or method listed is relevant to your study. If you are not sure if a list item applies to your research, read the appropriate section before selecting a response.

## Materials & experimental systems

| n/a | Involved in the study |
|---|---|
| ☒ | ☐ Antibodies |
| ☒ | ☐ Eukaryotic cell lines |
| ☒ | ☐ Palaeontology and archaeology |
| ☒ | ☐ Animals and other organisms |
| ☐ | ☒ Clinical data |
| ☒ | ☐ Dual use research of concern |
| ☒ | ☐ Plants |

## Methods

| n/a | Involved in the study |
|---|---|
| ☒ | ☐ ChIP-seq |
| ☒ | ☐ Flow cytometry |
| ☒ | ☐ MRI-based neuroimaging |

## Clinical data

Policy information about clinical studies

All manuscripts should comply with the ICMJE guidelines for publication of clinical research and a completed CONSORT checklist must be included with all submissions.

| | |
|---|---|
| Clinical trial registration | NCT05308576 |
| Study protocol | The protocol is available in the Supplementary materials. |
| Data collection | In this phase 3 study, participants were recruited from 9 study sites located across three provinces in China, including Sichuan, Guizhou, and Hunan. Between December 26, 2022 and January 15, 2023, a total of 11,010 participants were initially screened for eligibility. Of them, 9,223 individuals were randomized at a 1:1 ratio to receive either SCTV01E or placebo. |
| Outcomes | The primary endpoint of this study was cases of the first occurrence of symptomatic infection of SARS-CoV-2 of any severity starting 7 days post-vaccination. The second efficacy endpoints included: cases of the first occurrence of all infection (including asymptomatic infection), asymptomatic infection, obvious infection of SARS-CoV-2, moderate and above, severe and above COVID-19 and death due to COVID-19, respectively, starting 7 days post-vaccination; cases of the first occurrence of all infection (including asymptomatic infection), asymptomatic infection, symptomatic infection, and obvious infection of SARS-CoV-2, respectively, starting 14 days (≥15 days) post-vaccination; cases of the first occurrence of symptomatic infection, obvious infection of SARS-CoV-2, moderate and above, severe and above COVID-19 and death due to COVID-19, respectively, caused by SARS-CoV-2 variants and subvariants starting 14 days (≥15 days) post-vaccination.<br><br>The immunogenicity endpoints in the study included: GMT of neutralizing antibody (nAb) against SARS-CoV-2 variants or subvariants on 7, 14, 28, 90, 180 and 365 days post-vaccination; the sereresponse rates (SRRs) of nAb (change from <lower limit of quantification (LLOQ) to ≥4 ×LLOQ, or at least a fourfold rise if baseline ≥LLOQ) compared with the pre-injection baseline (95%CI) against SARS-CoV-2 variants or subvariants on 7, 14, 28, 90, 180 and 365 days post-vaccination.<br><br>The safety endpoints in the study included: the incidence and severity of solicited AEs of SCTV01E from Day 0 to Day 7; incidence and severity of unsolicited AEs from Day 0 to Day 28; incidence and severity of serious AEs and AESIs from Day 0 to Day 365. The severity of AEs was graded according to the criteria of the Toxicity Grading Scale for Healthy Adult and Adolescent Volunteers Enrolled in Preventive Vaccine Clinical Trials.<br><br>In the post hoc analysis, the viral load in a patient's sample was quantified using cycle threshold (CT) obtained from the RT-PCR test. The CT value indicates the number of amplification cycles needed for the SARS-CoV-2 RNA sequence to become detectable. To estimate the number of viral particles present in one milliliter of the sample being tested, the CT value was converted into log10 copies/mL using the formula 13.2 - CT* 0.30769. |
